# Hepatic Gene Expression Profiling in Nrf2 Knockout Mice after Long-Term High-Fat Diet-Induced Obesity

**DOI:** 10.1155/2013/340731

**Published:** 2013-04-18

**Authors:** Dionysios V. Chartoumpekis, Panos G. Ziros, Apostolos Zaravinos, Ralitsa P. Iskrenova, Agathoklis I. Psyrogiannis, Venetsana E. Kyriazopoulou, Gerasimos P. Sykiotis, Ioannis G. Habeos

**Affiliations:** ^1^Division of Endocrinology, Department of Internal Medicine, Medical School, University of Patras, 26504 Patras, Greece; ^2^Department of Pharmacology and Chemical Biology, School of Medicine, University of Pittsburgh, Pittsburgh, PA 15261, USA; ^3^Laboratory of Virology, Medical School, University of Crete, 71110 Heraklion, Greece; ^4^Department of Biological Sciences, Molecular Medicine Research Center and Laboratory of Molecular and Medical Genetics, University of Cyprus, 1678 Nicosia, Cyprus

## Abstract

*Introduction*. The transcription factor NFE2-related factor 2 (Nrf2) is a central regulator of antioxidant and detoxification gene expression in response to electrophilic or oxidative stress. Nrf2 has recently been shown to cross-talk with metabolic pathways, and its gene deletion protected mice from high-fat-diet-(HFD-) induced obesity and insulin resistance. This study aimed to identify potential Nrf2-regulated genes of metabolic interest by comparing gene expression profiles of livers of wild-type (WT) versus Nrf2 knockout (Nrf2-KO) mice after a long-term HFD. *Methods*. WT and Nrf2-KO mice were fed an HFD for 180 days; total RNA was prepared from liver and used for microarray analysis and quantitative real-time RT-PCR (qRT-PCR). *Results*. The microarray analysis identified 601 genes that were differentially expressed between WT and Nrf2-KO mice after long-term HFD. Selected genes, including ones known to be involved in metabolic regulation, were prioritized for verification by qRT-PCR: *Cyp7a1* and *Fabp5* were significantly overexpressed in Nrf2-KO mice; in contrast, *Car*, *Cyp2b10*, *Lipocalin 13*, *Aquaporin 8*, *Cbr3*, *Me1*, and *Nqo1* were significantly underexpressed in Nrf2-KO mice. *Conclusion*. Transcriptome profiling after HFD-induced obesity confirms that *Nrf2* is implicated in liver metabolic gene networks. The specific genes identified here may provide insights into Nrf2-dependent mechanisms of metabolic regulation.

## 1. Introduction

Obesity, type 2 diabetes, and the metabolic syndrome are multifactorial diseases [1] that are considered an epidemic in westernized societies [2]; by increasing the risk of cardiovascular events, cancer, and other diseases, they have detrimental effects on life expectancy and quality [3, 4]. Although knowledge on the pathophysiology of obesity and diabetes is expanding, the identification of new molecular pathways involved in these disorders is necessary to better understand their pathogenesis and to identify potential drug targets. A transcription factor that has recently been implicated in obesity and metabolic dysregulation is Nrf2 (NFE2-related factor 2), encoded by *NFE2L2* (nuclear erythroid factor 2-like 2) [5].

Nrf2 is a transcription factor of the “cap n'collar” family that has a central role in maintaining cellular homeostasis in response to oxidative and electrophilic stress [6–9]. Under basal conditions Nrf2 is localized mainly in the cytoplasm where it binds to the Kelch-like ECH-associating protein (Keap1) and is thereby targeted for ubiquitination and proteasomal degradation. Upon exposure to oxidative and electrophilic stress, Nrf2 escapes Keap1-mediated degradation and accumulates in the nucleus where it binds to *cis* elements in the regulatory domains (antioxidant response elements, AREs) of antioxidant and detoxification genes, inducing their expression [10].

Nrf2 has been described to have a protective function against a number of pathologies that are caused or aggravated by oxidative stress such as cancer, pulmonary disease, and neurodegenerative or inflammatory conditions [11, 12]. Recently, a role of Nrf2 in obesity has also been discovered. Using mainly the Nrf2 knockout (Nrf2-KO) mice as a model, it has been shown by our group and by others that deletion of *Nfe2l2* protected mice from diet-induced obesity and insulin resistance [13–16]. In these studies, a variety of diet types has been used: high-fat diet with 60 kcal% fat [13, 16], high-fat diet with 41 kcal% fat [14], and high-fat western diet with 39.7 kcal% fat [15]. The exact mechanisms underlying the protective effect of Nrf2 deletion in high-fat diet-induced obesity remain to be elucidated. However, there is evidence that the cross-talk of Nrf2 with other metabolic factors such as peroxisome proliferator-activated receptor gamma (PPAR*γ*) [14] or fibroblast growth factor 21 (FGF21) [13] may, at least partially, explain this phenotype. In a recent study, we described the phenotypic comparison of WT versus Nrf2-KO mice under high-fat diet (HFD, 60 kcal% fat) or a control diet (standard diet, St.D.) for 180 days [13]. Briefly, under St.D. no difference was observed in body weight gain, glucose tolerance, or insulin tolerance between the two genotypes. While under HFD, both genotypes initially gained weight at about the same rate, the Nrf2-KO mice reached a plateau earlier than WT, and after about 90 days on HFD weighed significantly lower than WT (about 15% lower). Already after 30 days on HFD, the Nrf2-KO mice were significantly more glucose tolerant than WT, and after 180 days they were also significantly more insulin sensitive (as evidenced by intraperitoneal (i.p.) glucose tolerance test and i.p. insulin tolerance test) [13].

Gene expression profiling studies in Nrf2-KO mice under metabolic stress have not yet been reported. The present study used microarray analysis to investigate hepatic genes and gene networks that are regulated directly or indirectly by Nrf2 in mice on a long-term (180 days) HFD regimen.

## 2. Materials and Methods

### 2.1. Mice

All animal procedures were approved by the institutional review board of the University of Patras Medical School and were in accordance with E.C. Directive 86/609/EEC. *C57BL6J Nrf*2^+/−^ mice, originally developed by Professor M. Yamamoto, were obtained from RIKEN BRC (Tsukuba, Japan). Wild type (WT) and Nrf2-KO mice were generated by mating *Nrf*2^+/−^ male and female mice; the offspring were genotyped as previously described [17]. Male WT and Nrf2-KO mice (9-10 weeks old) were fed *ad libitum* an St.D. (10 kcal% fat) or an HFD (60 kcal% fat, Research Diets, New Brunswick, NJ) for 180 days (*n* = 8 for each group). Mice were housed in the animal facility of the University of Patras Medical School in temperature-, light-, and humidity-controlled rooms with a 12 h light/dark cycle.

### 2.2. Liver RNA Isolation

Liver was excised from mice and was submerged immediately in RNA later solution (Ambion, Foster City, CA). Total RNA was isolated from liver samples from WT or Nrf2-KO mice using the TRIzol reagent (Life Technologies, Carlsbad, CA), following the manufacturer's instructions, and further purified using the RNeasy mini-kit (Qiagen, Hilden, Germany). RNA yield and quality were determined with a NanoDrop 1000 Spectrophotometer (NanoDrop Technologies, Montchanin, DE) and a 2100 Bioanalyzer (Agilent Technologies, Santa Clara, CA). For microarray analysis, pooled RNA from either WT or Nrf2-KO mice was used. For qRT-PCR purposes, RNA from individual liver samples was used to generate cDNA. 

### 2.3. Microarray Experimentation and Analysis

The microarray experiments were performed using total pooled RNA from liver samples of 8 WT or 8 Nrf2-KO mice fed an HFD for 180 days. Four technical replicates were used for each genotype. Microarray experiments were carried out as one-color hybridizations on murine 4plex arrays from Agilent. The Agilent Whole Mouse Genome Microarray 4x44K slides were used (8 slides in total), each slide including 39430 probes. The labelling reaction of total RNA was performed using the Low Input Quick Amp Labelling Kit (Agilent) using 100 ng of total RNA as starting material, according to the manufacturer's instructions. cRNA synthesis was regarded successful provided that ≥1.65 *μ*g of cRNA with a Cy3-incorporation rate ≥8.0 pmol/*μ*g cRNA were synthesized. Fragmentation and hybridization of cRNA was performed as follows: 1.65 *μ*g of Cy3-labelled cRNA were fragmented according to the manufacturer's instructions, and 1.425 *μ*g of fragmented cRNA were hybridized. The hybridization was performed at 65°C for 17 h in an Agilent hybridization oven. Agilent arrays were then washed, scanned, and processed according to the supplier's protocol. After scanning at 5 *μ*m resolution with a DNA microarray laser scanner (Agilent), features were extracted with image analysis tool version A.8.3.1 using default protocols and settings (Agilent). Primary data analysis was performed using Agilent's Feature Extraction Software (version 10.7.3.1). ATLAS Biolabs (Berlin, Germany) performed labelling and hybridization of samples as well as generation of the primary data.

The raw microarray data were initially background corrected, normalized using quantile normalization, and further log2 transformed. Significantly up- or downregulated genes were identified using Significance Analysis of Microarrays (SAM) in the software platform MeV 4.8 (TM4 Microarray Software Suite) [18, 19]. SAM assigns a score to each gene on the basis of a change in gene expression relative to the standard deviation of repeated measurements. For genes with scores greater than an adjustable threshold, SAM uses permutations of the repeated measurements to estimate the percentage of genes identified by chance (the false discovery rate, FDR). Analysis parameters (Delta) were set to result in FDR ≤ 1% (a stringent criterion).

### 2.4. GEO Accession Numbers

Microarray data discussed in this publication are MIAME compliant and have been deposited in NCBI gene expression omnibus with the following accession number: GSE33575 (GSM830131 through GSM830138). 

### 2.5. Gene Ontology (GO) and Enrichment Analysis

Gene ontology (GO) analysis is helpful for the deduction of conclusions from microarray data. GO is a database with curated annotations for known genes, that is, gene biological processes, molecular functions, and cellular components. GO analysis was performed, using the Genesis 1.7.2 software and the WebGestalt toolkit (http://bioinfo.vanderbilt.edu/webgestalt/), as previously reported [20, 21]. The hypergeometric test with Bonferroni correction was used for enrichment evaluation analysis. The *R* function adjP was used in order to adjust the nominal *P* values of the large number of categories at the same time. The significance level for the adjusted *P* value was set at 0.01, and the minimum number of genes for a category was set at 2. 

### 2.6. Ingenuity Pathway Analysis

Differentially expressed genes (DEGs) were investigated for network interrelation by ingenuity pathway analysis (IPA) software (Ingenuity Systems, Redwood City, CA). IPA scans the set of input genes to identify networks by using the ingenuity pathway knowledge base for interactions between identified “focus genes.” In this study, the liver DEGs between WT and Nrf2-KO mice and hypothetical interacting genes (stored in the knowledge base in IPA software) were used to generate a set of networks with a maximum network size of 35 genes/proteins. Networks are displayed graphically as genes/gene products (nodes) and the biological relationships between the nodes (edges). All edges are from canonical information stored in the ingenuity pathways knowledge base. In addition, IPA computes a score for each network according to the fit of the user's set of significant genes. The score indicates the likelihood that the focus genes in a network from ingenuity's knowledge base are found together due to random chance. A score of 3, as the cutoff for identifying gene networks, indicates that there is only a 1/1000 chance that the focus genes shown in a network are due to random chance; therefore, a score ≥3 indicates a 99.9% confidence level.

### 2.7. Quantitative Real-Time PCR

Total RNA from individual liver samples was used for cDNA synthesis after a DNAse digestion step (Turbo DNase, Life Technologies) so as to prevent genomic DNA contamination. cDNA was synthesized using the superscript first-strand synthesis system (Life Technologies), and quantitative real-time PCRs were performed in triplicate 20 *μ*L reaction volumes on a StepOnePlus Instrument (Applied Biosystems, Foster City, CA) using Fast SYBR Green Master Mix (Applied Biosystems). Relative mRNA levels were calculated by the comparative threshold cycle method using TBP (TATA-binding protein) as the housekeeping gene. PCR efficiency was determined from a standard curve, and the Pfaffl method was used to calculate fold changes [22]. The correct size of the PCR products was confirmed by electrophoresis on a 2.5% agarose gel stained with ethidium bromide. Purity of the amplified products was assessed by melting curve analysis using the StepOne Software version 2.1 (Applied Biosystems). The primers used for *Cyp7a1* (cytochrome P450, family 7, subfamily A, polypeptide 1),* Fabp5 *(fatty acid binding protein 5),* Car *(constitutive androstane receptor),* Cyp2b10 *(cytochrome P450, family 2, subfamily B, polypeptide 10), *Lipocalin 13, Aquaporin 8, Cbr3* (carbonyl reductase 3)*, Me1 *(malic enzyme 1), and *Nqo1* (NADPH dehydrogenase quinone 1) were obtained from the PrimerBank (Center for Computational and Integrative Biology, Harvard Medical School, Massachusetts, USA) [23–25]. All primer sequences are shown in Table S1 (see Supplementary Material available online at http://dx.doi.org/10.1155/2013/340731).

### 2.8. Statistical Analysis

In microarrays, normality of the data distribution was checked by the Kolmogorov-Smirnov test. Differences in gene expression levels between WT and Nrf2-KO mice in liver were explored using SAM and the *t*-test. Numerical values were expressed as the mean ± standard deviation (SD). Statistical significance was set at the 95% confidence level (*P* < 0.05), and the fold change cutoff was set at 2. For statistical analysis of qRT-PCR data, one-way ANOVA followed by Tukey's test was used; qRT-PCR data were expressed as the mean ± SD. The number of biological or technical replicates used is described in the corresponding results. Statistical significance was set at the 95% confidence level (*P* < 0.05). The statistical package GraphPad Prism was used for calculations (GraphPad Software, La Jolla, CA).

## 3. Results

### 3.1. Differentially Expressed Liver Genes between Nrf2-KO and WT Mice Fed an HFD for 180 Days

SAM analysis, based on strict statistical criteria (fold change >2; median FDR < 0.01; 90th percentile FDR = 0.32), identified 601 liver differentially expressed genes (DEGs) between Nrf2-KO and WT mice after 180 days on HFD. Of these genes, 428 were significantly overexpressed (Table S2) and 173 were significantly underexpressed (Table S3) in Nrf2-KO versus WT mice. The 601 DEGs were clustered using a two-dimensional hierarchical clustering with Euclidean distance. Figure S1 depicts the heatmap of the genes that were over- (Figure S1A) or underexpressed (Figure S1B) in Nrf2-KO versus WT mice.

### 3.2. Gene Ontology (GO) Analysis of Differentially Expressed Genes

To obtain insights into the functions of the 601 DEGs, gene ontology (GO) analysis was performed. The main processes that these genes are involved in are categorized as follows: (1) immune response; (2) inflammatory response; (3) carbohydrate and pattern binding; (4) G protein and chemokine receptor binding; (5) glutathione transferase activity; (6) peptidase inhibitor activity; (7) cell surface, plasma membrane, and extracellular region genes; and (8) ion homeostasis.  Table 1 lists the differentially expressed genes implicated in each of the aforementioned functions.

### 3.3. qRT-PCR Verification of Microarray Results

**Figure 1:**
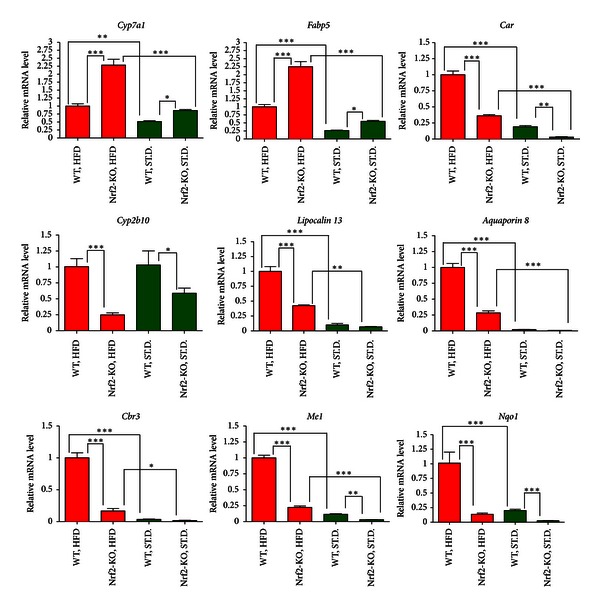
Relative mRNA levels based on qRT-PCR analysis. *Cyp7a1*, *Fabp5*, *Car*, *Cyp2b10*, *Lipocalin 13*, *Aquaporin8*, *Cbr3*, *Me1*, and *Nqo1* were selected for quantification with qRT-PCR in WT and Nrf2-KO mice under standard diet (St.D.) or high-fat diet (HFD) for 180 days (*n* = 8 for each genotype in either diet type). The qRT-PCR was performed in triplicate wells for each sample. Bars show means ± SD. *P < 0.01, **P < 0.001, ***P < 0.0001.

**Figure 2:**
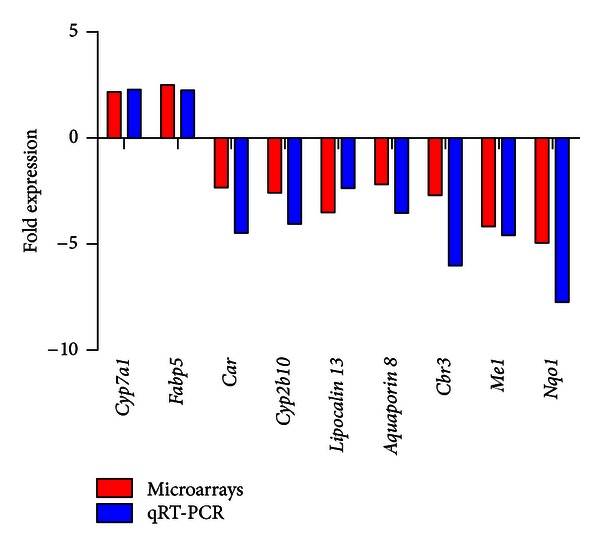
Correlation between microarray and qRT-PCR data. Nine genes identified by microarray analysis differentially expressed in the liver between Nrf2-KO and WT mice after 180 days on HFD were selected for qRT-PCR quantification. The red bars show the fold difference in the mRNA expression of a gene as calculated by microarray analysis, and the blue bars show the relevant fold difference as calculated by qRT-PCR analysis. Very good agreement was observed between the microarrays and qRT-PCR (Pearson correlation coefficient = 0.919; *P* = 0.0004).

For validation of the microarray data, a group of genes known to be directly or indirectly implicated in lipid or carbohydrate metabolism were selected for quantification with quantitative real-time RT-PCR (qRT-PCR). These genes were *Cyp7a1* (cytochrome P450, family 7, subfamily A, polypeptide 1), which is the rate limiting enzyme in bile acid synthesis from cholesterol;* Fabp5 *(fatty acid binding protein 5);* Car (Nr1I3) *(constitutive androstane receptor);* Cyp2b10 *(cytochrome P450,family 2, subfamily B, polypeptide 10), which is a *Car* target; *Lipocalin 13*;* Aquaporin 8*;* Cbr3* (carbonyl reductase 3); and *Me1 *(malic enzyme 1). *Nqo1* (NAD(P)H dehydrogenase, quinone 1) was selected as a prototypical *Nrf2* target gene. These genes were quantified not only in the liver of WT and Nrf2-KO mice under HFD for 180 days, but also in the liver of WT and Nrf2-KO mice under standard diet (St.D.) for the same time period. The relative gene expression levels are depicted in Figure 1. *Cyp7a1* and *Fabp5* were found to be over-expressed in the livers of Nrf2-KO mice after HFD feeding compared to WT mice, while the rest of the genes were under-expressed. There was excellent agreement between the microarray and the qRT-PCR data (Pearson correlation coefficient = 0.919; *P* value <0.001) (Figure 2).

### 3.4. Canonical Pathways and Networks Impacted by Nrf2 under HFD Feeding

Ingenuity pathway analysis (IPA) was used to rank gene networks by order of consistency of the microarray results with relationships confirmed by previously published results. Figure S2 presents a network which comprises Nrf2 and was generated by the microarray data. Nrf2 and genes that are under-expressed in the Nrf2-KO mice are shown in green; genes that are over-expressed in the Nrf2-KO mice are shown in red. It is obvious that all of the under-expressed genes in this network have been described to be directly regulated by Nrf2: carboxylesterase 1 g (*Ces1g*) [26]; glutathione S-transferase mu 5 (*Gstm5*)[27]; glutathione S-transferase alpha 5 (*Gsta5*) [28]; and NAD(P)H dehydrogenase quinone 1 (*Nqo1*) [29]. In contrast, none of the genes that are over-expressed in the Nrf2-KO mice is known to be directly regulated by Nrf2. These genes are calcitonin-related polypeptide beta (*Calcb*); collagen type V alpha 2 (*Col5a2*); cytochrome P450 family 2 subfamily C polypeptide 8 (*Cyp2c8*); growth factor independent 1 (*Gfi1*); H2-M2 histocompatibility 2, M region, locus 2 (*H2-m2*); interferon gamma (*Ifng*); solute carrier family 14 (urea transporter) member 1 (*Slc14a1*); solute carrier family 26 member 3 (*Slc26a3*); solute carrier family 9 (sodium hydrogen exchanger) member 3 (*Slc9a3*); serine peptidase inhibitor, Kazal type 4 (*Spink4*); sulfotransferase family 1E, estrogen preferring, member 1 (*Su1lte1*); and trefoil factor 1 (*Tff1*).

**Figure 3:**
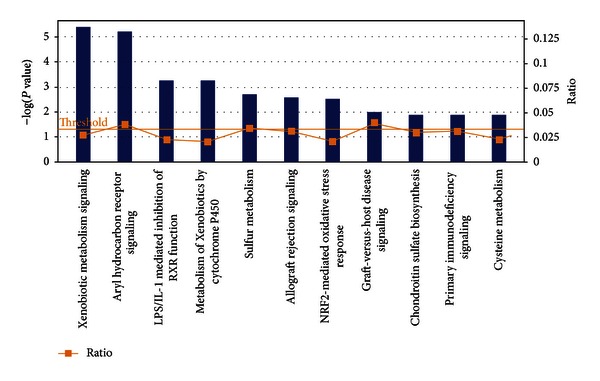
Significantly enriched canonical pathways identified by IPA. The diagram shows significantly overrepresented canonical pathways. A multiple testing corrected *P* value was calculated using the Benjamini-Hochberg method to control the rate of false discoveries in statistical hypothesis testing. The ratio value represents the number of molecules in a given pathway that meet cutoff criteria, divided by the total number of molecules associated with the respective biological function.

IPA analysis identified the statistically significant canonical pathways in the gene list. A corrected Fischer's exact test *P* value <0.05 was used as the threshold of significance (Figure 3). The number of genes (*n*) that were differentially expressed in each canonical pathway is shown below along with the *P* value and the ratio. In the “xenobiotic metabolism signaling” pathway (*n* = 7; *P* value = 3.95*E* − 06; ratio = 0.027), *Cyp2c8* and *Sult1e1* were over-expressed, whereas *Gstm5*, *Ces1g*, *Gsta5*, *Nqo1*, *Nfe2l2* (as expected), and sulfotransferase family cytosolic 2A dehydroepiandrosterone-preferring member 1 (*Sult2a1*) were under-expressed. In the “aryl hydrocarbon receptor signaling” pathway (*n* = 6; *P* value = 5.88*E* − 06; ratio = 0.038), *Tff1* and Fas ligand (*Faslg*) were over-expressed, whereas *Gstm5*, *Gsta5*, *Nqo1*, and *Nfe2l2* were underexpressed. In the “LPS/IL-1 mediated inhibition of RXR function” pathway (*n* = 5; *P* value = 5.49*E* − 04; ratio = 0.022), *Cyp2c8* and *Sult1e1* were over-expressed, whereas Gstm5, Gsta5, and Sult2a1 were underexpressed. In the “metabolism of xenobiotics by cytochrome P450” pathway (*n* = 4; *P* value = 5.65*E* − 04; ratio = 0.02), *Cyp2c8* was over-expressed and *Cyp2b13*/*Cyp2b9*, *Gstm5*, and *Gsta5* were underexpressed. In the “sulfur metabolism” pathway (*n* = 2; *P* value = 1.96*E* − 03; ratio = 0.034), *Sult1e1* was overexpressed, whereas *Sult2a1* was underexpressed.

## 4. Discussion

The findings of our previous study that male Nrf2-KO mice were at least partially protected from HFD-induced (60 kcal% fat) obesity and were more insulin sensitive and more glucose tolerant compared to their WT counterparts [13] are consistent with previous reports using comparable but different treatment parameters (40 kcal% fat diet or modified high-fat-diets) [14–16]. The purpose of this study was to identify hepatic genes differentially expressed between WT and Nrf2-KO mice after long-term (180 days) high-fat-diet-(HFD-) induced obesity. Such information could provide insights into the recently appreciated implication of Nrf2 in the development of obesity and metabolic syndrome. To this end, microarray-based transcriptome analysis was performed, employing strict statistical criteria.

The microarray-based gene expression analysis in these mice generated a total of 601 genes that were differentially expressed between the two genotypes: 478 genes were over-expressed in the Nrf2-KO mice, and 173 were underexpressed. These genes are not only implicated in functions that are typical of Nrf2 target genes, such as immune response [30], inflammation [31–33], and glutathione transferase activity [34, 35], but some gene groups were also found to be associated with carbohydrate and pattern binding (interacting selectively and noncovalently with a repeating or polymeric structure, such as a polysaccharide or peptidoglycan); G protein and chemokine receptor binding; peptidase inhibitor activity; cell surface, plasma membrane, and extracellular region genes; and ion homeostasis. These results further reinforce the notion that Nrf2 should not be regarded solely as a central antioxidant transcription factor, but it may be also implicated directly or indirectly in various tissue-specific homeostatic and/or physiological processes [11].

 Among a total 601 differentially expressed genes (DEGs), a subset was selected for validation by qRT-PCR quantification based on their relevance to metabolic pathways. The metabolic pathways and the respective representative genes chosen were bile acid synthesis from cholesterol (*Cyp7a1*), free fatty acid binding and transport (*Fabp5)*, glucose metabolism (*Lipocalin 13*), glycerol transport (*Aquaporin 8*), fatty acid biosynthesis (*Me1*), and energy homeostasis (*Car *and its target gene *Cyp2b10*). *Nqo1 *mRNA levels were quantified as *Nqo1* is considered a prototypical Nrf2 target gene.

The qRT-PCR-based mRNA quantification of specific genes of metabolic interest that were either over-expressed (*Cyp7a1* and *Fabp5*) or under-expressed (*Car*, *Cyp2b10*, *Lipocalin 13*, *Aquaporin 8*, *Cbr3*, and *Me1*) in Nrf2-KO mice compared to WT under HFD revealed potential candidate genes that may be implicated in the development of the different metabolic phenotype of Nrf2-KO mice. As shown in Figure 1, the differential expression of some of these genes was also evident under the St.D. regimen (with the exception of *Aquaporin 8*, *Lipocalin 13,* and *Cbr3*), indicating that these genes may be regulated by *Nrf2* under basal conditions as well. HFD for 180 days increased the expression of these genes (with the exception of *Cyp2b10* in both genotypes and of *Nqo1 *in the Nrf2-KO mice), and the fold difference between the two genotypes was generally accentuated. This observation may indicate that the possible regulation (direct or indirect) of these genes by *Nrf2* becomes more prominent under stress conditions (HFD-induced obesity). Given that *Nqo1 *is a prototypical Nrf2 target, its over-expression after HFD in WT mice suggests an increase in the transcriptional activity of Nrf2 by HFD, as supported by the fact that *Nqo1* induction was not observed in the Nrf2-KO mice under HFD. The possible functional importance of each of the other validated DEGs is discussed below.

*Cyp7a1* is the rate-limiting enzyme in bile acid synthesis from cholesterol. In agreement with previous studies [36], we show that *Cyp7a1* mRNA levels increased significantly in both genotypes after HFD feeding (Figure 1). The *Cyp7a1* mRNA levels also differed between the two genotypes, with the Nrf2-KO mice showing higher levels under St.D. (about 60% higher) and much higher levels under HFD (about 120% higher) compared to WT. In a previous study, a short-term (30 days) HFD did not accentuate the basal difference between the two genotypes [36], probably because an HFD feeding for a shorter period exposed the animals to lower metabolic and oxidative stress, such that the differences caused by Nrf2 deletion were not as pronounced. Moreover, given that small heterodimer partner (Shp) represses Cyp7a1 expression [37], and Nrf2 induces *Shp* gene expression [38], a reasonable hypothesis could be that Nrf2 represses *Cyp7a1* expression through *Shp*. This repression of *Cyp7a1* expression is abrogated by *Nrf2* deletion, leading to increased levels of *Cyp7a1* in Nrf2-KO mice compared to WT, which may partially protect them from obesity [39]. To clarify these molecular mechanisms, future experiments should involve *Shp* and *Nrf2* single and double KO mice. 

*Fabp5* is a member of the fatty acid-binding proteins which binds free fatty acids and regulates lipid metabolism and transport; it was first identified as being upregulated in psoriasis tissue [40]. In this study, *Fabp5* was increased after HFD feeding in both genotypes (Figure 1), which is in agreement with previous studies that have shown strong up-regulation of Fabp5 by western-type diet or HFD [41, 42]. *Fabp5* also exhibited higher mRNA levels in Nrf2-KO mice under either St.D. or HFD; a study using proteomic analysis showed similar results [43]. The specific physiological significance of *Fabp5* elevation in Nrf2-KO mice remains to be elucidated. Further experiments with Nrf2 over-expression or silencing and with concurrent measurement of Nrf2 levels in hepatocytes are warranted to elucidate the possible regulation of *Fabp5* by Nrf2.

*Car *(*Nr1I3*), initially characterized as a sensor of xenobiotics that regulates responses to toxicants [44], has recently been implicated in the control of energy and metabolism [45]. *Car* has been ascribed a function as an antiobesity receptor, because treatment of mice with a Car agonist partially prevented HFD-induced obesity in mice, and partially reversed obesity in mice that were already obese [46, 47]. In the present study, mRNA levels of *Car*, along with those of its primary target gene, *Cyp2b10* [48], were lower in Nrf2-KO mice compared to WT under either St.D. or HFD (Figure 1). In this case, the lower expression of Car cannot justify the ameliorated metabolic phenotype of Nrf2-KO compared to WT after long-term HFD. Nevertheless, the observation that *Car* and *Cyp2b10* mRNA levels are lower in Nrf2-KO mice than WT is in accordance with previous studies [49, 50]. Car mRNA was found to be increased in both genotypes after the HFD regimen. But *Cyp2b10* that is considered a *Car* target gene does not follow the same trend. This may indicate a difference in the mRNA turnover of *Cyp2b10* that may or may not be reflected in the protein levels. Further investigations, such as cell culture studies with manipulation of *Nrf2* levels/activity and measurement of *Car* levels/activity, are necessary to clarify the mechanisms that underlie the possible regulation of *Car* by *Nrf2*. 

*Lipocalin 13* is a lipocalin family member involved in glucose metabolism, and its deficiency is associated with obesity [51]. Herein, *lipocalin 13* liver mRNA levels were found to be lower in Nrf2-KO mice than in WT after long-term HFD; no difference was observed between the two genotypes under St.D. (Figure 1). As the existing data on the role of lipocalin 13 in obesity are scarce, this differential *lipocalin 13* mRNA expression between the two genotypes after the HFD feeding for 180 days warrants further elucidation.

Aquaporin family members are mainly water channels, but some of them have also been found to transport glycerol and to be involved in the development of obesity [52]. *Aquaporin 8* is expressed in liver [53], and in the present experimental model it exhibited lower mRNA expression in the liver of Nrf2-KO mice compared to WT after HFD. No difference was found between the two genotypes under St.D., but *aquaporin 8* was markedly induced in both genotypes after HFD with its levels being lower in the Nrf2-KO mice (Figure 1). As aquaporins may be implicated in the transport of glycerol (a product of the catabolism of triacylglycerols), a possible indirect regulation of aquaporin 8 by *Nrf2* may be of metabolic interest. 

*Cbr3 *catalyzes the reduction of carbonyl compounds (highly reactive lipid aldehydes) to the corresponding alcohols (inactive compounds) [54]. A recent clinical study [55] showed that a genetic variation in Cbr3 gene in humans correlates with type 2 diabetes and this effect can potentially be attributed to the catalysis of the conversion of prostaglandin E2 to prostaglandin F2*α*. In the present study, Cbr3 liver mRNA levels were about 5 times lower in Nrf2-KO mice after HFD compared to WT. Although WT mice tended to have greater *Cbr3* mRNA levels under standard diet, this difference was not statistically significant. Given that recent studies have shown that the *Cbr3* promoter comprises antioxidant response element (ARE) sequences that are recognized by Nrf2 to induce *Cbr3 *expression [50, 56, 57], it would be interesting to test whether the Nrf2-regulated *Cbr3* expression can contribute to the observed phenotype in the Nrf2-KO mice after HFD feeding. 

*Me1* is an enzyme that generates NADPH for fatty acid biosynthesis. In this study, *Me1* showed decreased mRNA levels in Nrf2-KO mice under St.D. or HFD; this is consistent with previous gene expression profiling studies that describe *Me1* as a Nrf2-dependent gene [58–60]. It has been previously shown that *Nrf2 *can redirect glucose and glutamine to anabolic pathways in cancer cells, and *Me-1* is implicated in these pathways [60]; however, these results in cancer cells cannot necessarily be safely extrapolated to nontransformed hepatocytes [61]. 

A limitation of this study is that microarray analysis was performed only in mice under HFD and not also on standard diet (St.D.). Thus, it is not possible to delineate among the 601 DEGs those genes that are differentially expressed irrespective of the treatment versus those that demonstrate diet-induced differential expression, except for the subset of genes that were validated by qRT-PCR, which analyzed gene expression in mice under both St.D. and HFD. Another limitation of this study is that the hepatic gene expression in this whole body knock-out model may be affected indirectly by endocrine factors that are secreted from other tissues (e.g., adipose tissue, muscle) that are also deficient in Nrf2. Therefore, some of the differentially expressed genes we detect in the liver may be affected by extrahepatic factors. The use of a liver-specific knock-out model could resolve this issue.

## 5. Conclusions

In conclusion, the current study showed that *Nrf2* deletion significantly altered the hepatic gene expression profile after long-term HFD, yielding a set of 601 DEGs that can be the focus of further studies on the role of *Nrf2* in obesity. The majority of these DEGs are involved in pathways relevant to the defense against oxidative and electrophilic stress, already known to be regulated by Nrf2. However, certain genes such as *Cyp7a1*, *Fabp5*, *Car*, *Cbr3,* and *Me1* can have specific metabolic effects and appear to be directly or indirectly regulated by *Nrf2*; these genes may be implicated in the less obese and more insulin sensitive metabolic phenotype of the Nrf2-KO mice. Novel mechanistic understanding and therapeutic interventions for obesity/metabolic syndrome may arise from the elucidation of the cross-talk of Nrf2 with metabolic pathways regulated by these genes.

## Supplementary Material

The supplementary material comprises of two figures and three tables. A heatmap of the differentially expressed genes between WT and Nrf2-KO mice after HFD feeding is depicted in figure S1. Figure S2 shows a gene network of some of the differentially expressed genes that also comprises Nrf2. The primer sequences used in this study are provided in Table S1. The 428 genes that were over-expressed as well the 173 genes that were under-expressed in the liver of the Nrf2-KO mice compared to their WT counterparts after 180 days on HFD, are presented in Tables S2 and S3 correspondingly.

## Figures and Tables

**Table 1:** Differentially expressed genes implicated in the enriched gene ontology processes.

Biological process—defense response—GO:0006952	
adj *P* = 0.0001	
Nupr1	Nuclear protein 1
Aoah	Acyloxyacyl hydrolase
Cd28	CD28 antigen
C8b	Complement component 8, beta polypeptide
Lat	Linker for activation of T cells
Calca	Calcitonin/calcitonin-related polypeptide, alpha
Prg2	Proteoglycan 2, bone marrow
Orm3	Orosomucoid 3
Clec2h	C-type lectin domain family 2, member h
Camp	Cathelicidin antimicrobial peptide
Wfdc12	WAP four-disulfide core domain 12
Chi3l3	Chitinase 3-like 3
Ccl5	Chemokine (C-C motif) ligand 5
Cd24a	CD24a antigen
Cfd	Complement factor D (adipsin)
Ccl12	Chemokine (C-C motif) ligand 12
Wfdc15b	WAP four-disulfide core domain 15B
Penk	Preproenkephalin
Sh2d1a	SH2 domain protein 1A
Ccl4	Chemokine (C-C motif) ligand 4
Mpo	Myeloperoxidase
Ccl8	Chemokine (C-C motif) ligand 8
Hamp	Hepcidin antimicrobial peptide
Hamp2	Hepcidin antimicrobial peptide 2
Cxcl9	Chemokine (C-X-C motif) ligand 9
Adora1	Adenosine A1 receptor

Biological process—immune response—GO:0006955	
adj *P* = 0.0001	
Cd8b1	CD8 antigen, beta chain 1
Thy1	Thymus cell antigen 1, theta
Ltb	Lymphotoxin B
Eomes	Eomesodermin homolog (Xenopus laevis)
Cd28	CD28 antigen
Igj	Immunoglobulin joining chain
H2-Aa	Histocompatibility 2, class II antigen A, alpha
Themis	Thymocyte selection associated
C8b	Complement component 8, beta polypeptide
Lat	Linker for activation of T cells
Cd8a	CD8 antigen, alpha chain
Prg2	Proteoglycan 2, bone marrow
Fasl	Fas ligand (TNF superfamily, member 6)
Lck	Lymphocyte protein tyrosine kinase
Ccl5	Chemokine (C-C motif) ligand 5
Cd24a	CD24a antigen
Btla	B and T lymphocyte associated
Cfd	Complement factor D (adipsin)
Ccl12	Chemokine (C-C motif) ligand 12
Bcl2a1d	B-cell leukemia/lymphoma 2 related protein A1d
Sh2d1a	SH2 domain protein 1A
Ccl4	chemokine (C-C motif) ligand 4
Mpo	Myeloperoxidase
Ccl8	Chemokine (C-C motif) ligand 8
Cxcl9	Chemokine (C-X-C motif) ligand 9
Serpina3g	Serine (or cysteine) peptidase inhibitor, clade A, member 3G

Biological process—immune system process—GO:0002376	
adj *P* = 0.0005	
Cd8b1	CD8 antigen, beta chain 1
Egr1	Early growth response 1
S100a9	S100 calcium binding protein A9 (calgranulin B)
Thy1	Thymus cell antigen 1, theta
Ltb	Lymphotoxin B
Cd28	CD28 antigen
Cd8a	CD8 antigen, alpha chain
Prg2	Proteoglycan 2, bone marrow
Fasl	Fas ligand (TNF superfamily, member 6)
Orm3	Orosomucoid 3
Lck	Lymphocyte protein tyrosine kinase
Ccl5	Chemokine (C-C motif) ligand 5
Btla	B and T lymphocyte associated
Cd24a	CD24a antigen
Ccl12	Chemokine (C-C motif) ligand 12
Ccl4	Chemokine (C-C motif) ligand 4
Bcl11b	B-cell leukemia/lymphoma 11B
Cd34	CD34 antigen
Cxcl9	Chemokine (C-X-C motif) ligand 9
Adora1	Adenosine A1 receptor
Eomes	Eomesodermin homolog (Xenopus laevis)
H2-Aa	Histocompatibility 2, class II antigen A, alpha
Igj	Immunoglobulin joining chain
Themis	Thymocyte selection associated
C8b	Complement component 8, beta polypeptide
Lat	Linker for activation of T cells
Cd5	CD5 antigen
Cfd	Complement factor D (adipsin)
Bcl2a1d	B-cell leukemia/lymphoma 2 related protein A1d
Sh2d1a	SH2 domain protein 1A
Mpo	Myeloperoxidase
Ccl8	Chemokine (C-C motif) ligand 8
Serpina3g	Serine (or cysteine) peptidase inhibitor, clade A, member 3G
Cd3d	CD3 antigen, delta polypeptide

Biological process—inflammatory response—GO:0006954	
adj *P* = 0.0010	
Nupr1	Nuclear protein 1
Aoah	Acyloxyacyl hydrolase
Cd28	CD28 antigen
C8b	Complement component 8, beta polypeptide
Lat	Linker for activation of T cells
Calca	Calcitonin/calcitonin-related polypeptide, alpha
Orm3	Orosomucoid 3
Chi3l3	Chitinase 3-like 3
Ccl5	Chemokine (C-C motif) ligand 5
Cd24a	CD24a antigen
Cfd	Complement factor D (adipsin)
Ccl12	Chemokine (C-C motif) ligand 12
Ccl4	Chemokine (C-C motif) ligand 4
Ccl8	Chemokine (C-C motif) ligand 8
Cxcl9	Chemokine (C-X-C motif) ligand 9
Adora1	Adenosine A1 receptor

Biological process—cellular di-, tri-valent inorganic cation homeostasis—GO:0030005	
adj *P* = 0.0035	
Lck	Lymphocyte protein tyrosine kinase
Cd24a	CD24a antigen
Ltf	Lactotransferrin
Gpr12	G-protein coupled receptor 12
Mt2	Metallothionein 2
Hamp	Hepcidin antimicrobial peptide
Hamp2	Hepcidin antimicrobial peptide 2
Mfi2	Antigen p97 (melanoma associated) identified by monoclonal antibodies 133.2 and 96.5
Calca	Calcitonin/calcitonin-related polypeptide, alpha
Slc39a5	Solute carrier family 39 (metal ion transporter), member 5

Biological process—cellular cation homeostasis—GO:0030003	
adj *P* = 0.0054	
Lck	Lymphocyte protein tyrosine kinase
Cd24a	CD24a antigen
Ltf	Lactotransferrin
Gpr12	G-protein coupled receptor 12
Mt2	Metallothionein 2
Hamp	Hepcidin antimicrobial peptide
Hamp2	Hepcidin antimicrobial peptide 2
Mfi2	Antigen p97 (melanoma associated) identified by monoclonal antibodies 133.2 and 96.5
Calca	Calcitonin/calcitonin-related polypeptide, alpha
Slc39a5	Solute carrier family 39 (metal ion transporter), member 5

Biological process—response to wounding—GO:0009611	
adj *P* = 0.0054	
Slc1a3	Solute carrier family 1 (glial high affinity glutamate transporter), member 3
Nupr1	Nuclear protein 1
Aoah	Acyloxyacyl hydrolase
Cd28	CD28 antigen
C8b	Complement component 8, beta polypeptide
Lat	Linker for activation of T cells
Calca	Calcitonin/calcitonin-related polypeptide, alpha
Tff1	Trefoil factor 1
Orm3	Orosomucoid 3
Chi3l3	Chitinase 3-like 3
Ccl5	Chemokine (C-C motif) ligand 5
Cd24a	CD24a antigen
Cfd	Complement factor D (adipsin)
Ccl12	Chemokine (C-C motif) ligand 12
Ccl4	Chemokine (C-C motif) ligand 4
Ccl8	Chemokine (C-C motif) ligand 8
Cxcl9	Chemokine (C-X-C motif) ligand 9
Adora1	Adenosine A1 receptor

Biological process—di-, tri-valent inorganic cation homeostasis—GO:0055066	
adj *P* = 0.0054	
Lck	Lymphocyte protein tyrosine kinase
Cd24a	CD24a antigen
Ltf	Lactotransferrin
Gpr12	G-protein coupled receptor 12
Mt2	Metallothionein 2
Hamp	Hepcidin antimicrobial peptide
Hamp2	Hepcidin antimicrobial peptide 2
Mfi2	Antigen p97 (melanoma associated) identified by monoclonal antibodies 133.2 and 96.5
Calca	Calcitonin/calcitonin-related polypeptide, alpha
Slc39a5	Solute carrier family 39 (metal ion transporter), member 5

Biological process—T cell activation—GO:0042110	
adj *P* = 0.0067	
Lck	Lymphocyte protein tyrosine kinase
Egr1	Early growth response 1
Cd5	CD5 antigen
Btla	B and T lymphocyte associated
Cd24a	CD24a antigen
Bcl2a1d	B-cell leukemia/lymphoma 2 related protein A1d
Cd28	CD28 antigen
H2-Aa	Histocompatibility 2, class II antigen A, alpha
Themis	Thymocyte selection associated
Bcl11b	B-cell leukemia/lymphoma 11B
Cd3d	CD3 antigen, delta polypeptide
Cd8a	CD8 antigen, alpha chain

Biological process—defense response to fungus—GO:0050832	
adj *P* = 0.0067	
Hamp	Hepcidin antimicrobial peptide
Hamp2	Hepcidin antimicrobial peptide 2
Mpo	Myeloperoxidase

Biological process—T cell differentiation—GO:0030217	
adj *P* = 0.0067	
Lck	Lymphocyte protein tyrosine kinase
Egr1	Early growth response 1
Bcl2a1d	B-cell leukemia/lymphoma 2 related protein A1d
Cd28	CD28 antigen
H2-Aa	Histocompatibility 2, class II antigen A, alpha
Themis	Thymocyte selection associated
Bcl11b	B-cell leukemia/lymphoma 11B
Cd3d	CD3 antigen, delta polypeptide
Cd8a	CD8 antigen, alpha chain

Molecular function—carbohydrate binding—GO:0030246	
adj *P* = 0.0029	
Vcan	Versican
Klrg1	Killer cell lectin-like receptor subfamily G, member 1
Ltf	Lactotransferrin
Gpnmb	Glycoprotein (transmembrane) nmb
Reg1	Regenerating islet-derived 1
Lgals2	Lectin, galactose-binding, soluble 2
Sftpd	Surfactant associated protein D
Prg2	Proteoglycan 2, bone marrow
Clec2h	C-type lectin domain family 2, member h
Chi3l3	Chitinase 3-like 3
Cd24a	CD24a antigen
Ncam1	Neural cell adhesion molecule 1
Abp1	Amiloride binding protein 1 (amine oxidase, Copper-containing)
Mpo	Myeloperoxidase
Klrd1	Killer cell lectin-like receptor, subfamily D, member 1
Crispld2	Cysteine-rich secretory protein LCCL domain containing 2
Ccl8	Chemokine (C-C motif) ligand 8

Molecular function—peptidase inhibitor activity—GO:0030414	
adj *P* = 0.0029	
Wfdc12	WAP four-disulfide core domain 12
Serpina9	Serine (or cysteine) peptidase inhibitor, clade A (alpha-1 antiproteinase, antitrypsin), member 9
Serpinb1a	Serine (or cysteine) peptidase inhibitor, clade B, member 1a
Bcl2a1d	B-cell leukemia/lymphoma 2 related protein A1d
Wfdc2	WAP four-disulfide core domain 2
Wfdc15b	WAP four-disulfide core domain 15B
Cst7	Cystatin F (leukocystatin)
Spink4	Serine peptidase inhibitor, Kazal type 4
Serpina3g	Serine (or cysteine) peptidase inhibitor, clade A, member 3G
Spink3	Serine peptidase inhibitor, Kazal type 3
Serpina12	Serine (or cysteine) peptidase inhibitor, clade A (alpha-1 antiproteinase, antitrypsin), member 12

Molecular function—glutathione transferase activity—GO:0004364	
adj *P* = 0.0032	
Gstt3	Glutathione S-transferase, theta 3
Gstm1	Glutathione S-transferase, mu 1
Gstm4	Glutathione S-transferase, mu 4
Gsta2	Glutathione S-transferase, alpha 2 (Yc2)
Gstm3	Glutathione S-transferase, mu 3

Molecular function—endopeptidase inhibitor activity—GO:0004866	
adj *P* = 0.0032	
Wfdc12	WAP four-disulfide core domain 12
Serpina9	Serine (or cysteine) peptidase inhibitor, clade A (alpha-1 antiproteinase, antitrypsin), member 9
Serpinb1a	Serine (or cysteine) peptidase inhibitor, clade B, member 1a
Bcl2a1d	B-cell leukemia/lymphoma 2 related protein A1d
Wfdc2	WAP four-disulfide core domain 2
Cst7	Cystatin F (leukocystatin)
Spink4	Serine peptidase inhibitor, Kazal type 4
Serpina3g	Serine (or cysteine) peptidase inhibitor, clade A, member 3G
Spink3	Serine peptidase inhibitor, Kazal type 3
Serpina12	Serine (or cysteine) peptidase inhibitor, clade A (alpha-1 antiproteinase, antitrypsin), member 12

Molecular function—G-protein-coupled receptor binding—GO:0001664	
adj *P* = 0.0033	
Ccl8	Chemokine (C-C motif) ligand 8
Ccl5	Chemokine (C-C motif) ligand 5
Cxcl9	Chemokine (C-X-C motif) ligand 9
Adrb3	Adrenergic receptor, beta 3
Ccl12	Chemokine (C-C motif) ligand 12
Calca	Calcitonin/calcitonin-related polypeptide, alpha
Ccl4	Chemokine (C-C motif) ligand 4

Molecular function—serine-type endopeptidase inhibitor activity—GO:0004867	
adj *P* = 0.0065	
Wfdc12	WAP four-disulfide core domain 12
Serpina9	Serine (or cysteine) peptidase inhibitor, clade A (alpha-1 antiproteinase, antitrypsin), member 9
Serpinb1a	Serine (or cysteine) peptidase inhibitor, clade B, member 1a
Serpina3g	Serine (or cysteine) peptidase inhibitor, clade A, member 3G
Spink4	Serine peptidase inhibitor, Kazal type 4
Spink3	Serine peptidase inhibitor, Kazal type 3
Serpina12	Serine (or cysteine) peptidase inhibitor, clade A (alpha-1 antiproteinase, antitrypsin), member 12
Wfdc2	WAP four-disulfide core domain 2

Molecular function—chemokine activity—GO:0008009	
adj *P* = 0.0097	
Ccl8	Chemokine (C-C motif) ligand 8
Ccl5	Chemokine (C-C motif) ligand 5
Cxcl9	Chemokine (C-X-C motif) ligand 9
Ccl12	Chemokine (C-C motif) ligand 12
Ccl4	Chemokine (C-C motif) ligand 4

Molecular function—chemokine receptor binding—GO:0042379	
adj *P* = 0.0097	
Ccl8	Chemokine (C-C motif) ligand 8
Ccl5	Chemokine (C-C motif) ligand 5
Cxcl9	Chemokine (C-X-C motif) ligand 9
Ccl12	Chemokine (C-C motif) ligand 12
Ccl4	Chemokine (C-C motif) ligand 4

Molecular function—pattern binding—GO:0001871	
adj *P* = 0.0097	
Vcan	Versican
Chi3l3	Chitinase 3-like 3
Ncam1	Neural cell adhesion molecule 1
Ltf	Lactotransferrin
Gpnmb	Glycoprotein (transmembrane) nmb
Abp1	Amiloride binding protein 1 (amine oxidase, copper-containing)
Crispld2	Cysteine-rich secretory protein LCCL domain containing 2
Mpo	Myeloperoxidase
Ccl8	Chemokine (C-C motif) ligand 8

Molecular function—polysaccharide binding—GO:0030247	
adj *P* = 0.0097	
Vcan	Versican
Chi3l3	Chitinase 3-like 3
Ncam1	Neural cell adhesion molecule 1
Ltf	Lactotransferrin
Gpnmb	Glycoprotein (transmembrane) nmb
Abp1	Amiloride binding protein 1 (amine oxidase, copper-containing)
Crispld2	Cysteine-rich secretory protein LCCL domain containing 2
Mpo	Myeloperoxidase
Ccl8	Chemokine (C-C motif) ligand 8

Cellular component—extracellular region—GO:0005576	
adj *P* = 5.75*e* − 09	
Col5a2	Collagen, type V, alpha 2
Vcan	Versican
Slc1a3	Solute carrier family 1 (glial high affinity glutamate transporter), member 3
Dmbt1	Deleted in malignant brain tumors 1
Ltb	Lymphotoxin B
Itgbl1	Integrin, beta-like 1
Mgp	Matrix Gla protein
Cst7	Cystatin F (leukocystatin)
Fgl2	Fibrinogen-like protein 2
Gzma	Granzyme A
Prom1	Prominin 1
Ltbp2	Latent transforming growth factor beta binding protein 2
Afp	Alpha fetoprotein
Calca	Calcitonin/calcitonin-related polypeptide, alpha
Tff1	Trefoil factor 1
Fasl	Fas ligand (TNF superfamily, member 6)
Camp	Cathelicidin antimicrobial peptide
Orm3	Orosomucoid 3
Ccl5	Chemokine (C-C motif) ligand 5
Ccl12	Chemokine (C-C motif) ligand 12
Abp1	Amiloride binding protein 1 (amine oxidase, copper-containing)
Penk	Preproenkephalin
Wfdc15b	WAP four-disulfide core domain 15B
Ccl4	Chemokine (C-C motif) ligand 4
Adipoq	Adiponectin, C1Q and collagen domain containing
Hamp	Hepcidin antimicrobial peptide
Cxcl9	Chemokine (C-X-C motif) ligand 9
Il17rb	Interleukin 17 receptor B
Mmp8	Matrix metallopeptidase 8
Svep1	Sushi, von Willebrand factor type A, EGF and pentraxin domain containing 1
Chi3l1	Chitinase 3-like 1
Gcg	Glucagon
Serpina9	Serine (or cysteine) peptidase inhibitor, clade A (alpha-1 antiproteinase, antitrypsin), member 9
Oosp1	Oocyte secreted protein 1
Ltf	Lactotransferrin
Sema3g	Sema domain, immunoglobulin domain (Ig), short basic domain, secreted, (semaphorin) 3G
Wfdc2	WAP four-disulfide core domain 2
C8b	Complement component 8, beta polypeptide
Lcn13	Lipocalin 13
Esm1	Endothelial cell-specific molecule 1
Dkk4	Dickkopf homolog 4 (Xenopus laevis)
Gm128	Predicted gene 128
Sftpd	Surfactant associated protein D
Muc4	Mucin 4
Chi3l3	Chitinase 3-like 3
Wfdc12	WAP four-disulfide core domain 12
Tff2	Trefoil factor 2 (spasmolytic protein 1)
Tff3	Trefoil factor 3, intestinal
Cpxm2	Carboxypeptidase X 2 (M14 family)
Cfd	Complement factor D (adipsin)
Nts	Neurotensin
Crispld2	Cysteine-rich secretory protein LCCL domain containing 2
Cd2	CD2 antigen
Mmp7	Matrix metallopeptidase 7
Ccl8	Chemokine (C-C motif) ligand 8
Cgref1	Cell growth regulator with EF hand domain 1
Hamp2	Hepcidin antimicrobial peptide 2
Gpc1	Glypican 1
Dpt	Dermatopontin
Mfi2	Antigen p97 (melanoma associated) identified by monoclonal antibodies 133.2 and 96.5
Spink4	Serine peptidase inhibitor, Kazal type 4
Spink3	Serine peptidase inhibitor, Kazal type 3
Serpina12	Serine (or cysteine) peptidase inhibitor, clade A (alpha-1 antiproteinase, antitrypsin), member 12
Fam3b	Family with sequence similarity 3, member B

Cellular component—plasma membrane—GO:0005886	
adj *P* = 1.09*e* − 07	
Cd8b1	CD8 antigen, beta chain 1
Thy1	Thymus cell antigen 1, theta
Adrb3	Adrenergic receptor, beta 3
Sirpb1	Signal-regulatory protein beta 1
Cd28	CD28 antigen
Treh	Trehalase (brush-border membrane glycoprotein)
Cd8a	CD8 antigen, alpha chain
Slc39a5	Solute carrier family 39 (metal ion transporter), member 5
Klrc1	Killer cell lectin-like receptor subfamily C, member 1
Clec2h	C-type lectin domain family 2, member h
Gpr68	G protein-coupled receptor 68
Gpr171	G protein-coupled receptor 171
Btla	B and T lymphocyte associated
Cd24a	CD24a antigen
Krt19	Keratin 19
Lair1	Leukocyte-associated Ig-like receptor 1
Gldn	Gliomedin
Gpr110	G protein-coupled receptor 110
Plxdc2	Plexin domain containing 2
Adora1	Adenosine A1 receptor
Muc3	Mucin 3, intestinal
Dsg1c	Desmoglein 1 gamma
Il17rb	Interleukin 17 receptor B
Mas1	MAS1 oncogene
Abcc3	ATP-binding cassette, sub-family C (CFTR/MRP), member 3
Lor	Loricrin
Gbp5	Guanylate binding protein 5
H2-Aa	Histocompatibility 2, class II antigen A, alpha
Espn	Espin
Glycam1	Glycosylation dependent cell adhesion molecule 1
Cd3g	CD3 antigen, gamma polypeptide
Lat	Linker for activation of T cells
Ildr1	Immunoglobulin-like domain containing receptor 1
Cxcr6	Chemokine (C-X-C motif) receptor 6
Plek2	Pleckstrin 2
Gpr65	G-protein coupled receptor 65
Aqp8	Aquaporin 8
Kcnn4	Potassium intermediate/small conductance calcium-activated channel, subfamily N, member 4
Slco1a1	Solute carrier organic anion transporter family, member 1a1
Pdcd1	Programmed cell death 1
Mapt	Microtubule-associated protein tau
Cd5	CD5 antigen
Clstn3	Calsyntenin 3
Gpr12	G-protein coupled receptor 12
Cd2	CD2 antigen
Gbp1	Guanylate binding protein 1
Vsig2	V-set and immunoglobulin domain containing 2
Gpc1	Glypican 1
Gna14	Guanine nucleotide binding protein, alpha 14
Slc1a3	Solute carrier family 1 (glial high affinity glutamate transporter), member 3
Itgbl1	Integrin, beta-like 1
Ltb	Lymphotoxin B
Arhgap10	Rho GTPase activating protein 10
Gbp2	Guanylate binding protein 2
Prom1	Prominin 1
Car9	Carbonic anhydrase 9
Pip5k1a	Phosphatidylinositol-4-phosphate 5-kinase, type 1 alpha
Rab25	RAB25, member RAS oncogene family
Fasl	Fas ligand (TNF superfamily, member 6)
Tjp3	Tight junction protein 3
Lck	Lymphocyte protein tyrosine kinase
Gpr174	G protein-coupled receptor 174
Slc16a13	Solute carrier family 16 (monocarboxylic acid transporters), member 13
Gnat2	Guanine nucleotide binding protein, alpha transducing 2
Ncam1	Neural cell adhesion molecule 1
Igsf9	Immunoglobulin superfamily, member 9
Klrd1	Killer cell lectin-like receptor, subfamily D, member 1
Art2b	ADP-ribosyltransferase 2b
Dsg1b	Desmoglein 1 beta
Cd34	CD34 antigen
Oscp1	Organic solute carrier partner 1
Itk	IL2-inducible T-cell kinase
Gpr128	G protein-coupled receptor 128
Rasgrp2	RAS, guanyl releasing protein 2
Slc26a3	Solute carrier family 26, member 3
Cdh1	Cadherin 1
Il2rb	Interleukin 2 receptor, beta chain
Gpnmb	Glycoprotein (transmembrane) nmb
Ms4a4b	Membrane-spanning 4-domains, subfamily A, member 4B
C8b	Complement component 8, beta polypeptide
Grin3b	Glutamate receptor, ionotropic, NMDA3B
Clic6	Chloride intracellular channel 6
Grm8	Glutamate receptor, metabotropic 8
Mfi2	Antigen p97 (melanoma associated) identified by monoclonal antibodies 133.2 and 96.5
Cd3d	CD3 antigen, delta polypeptide
Slc30a10	Solute carrier family 30, member 10
Pcdh20	Protocadherin 20

Cellular component—cell surface—GO:0009986	
adj *P* = 4.72*e* − 07	
Cd8b1	CD8 antigen, beta chain 1
Slc1a3	Solute carrier family 1 (glial high affinity glutamate transporter), member 3
Thy1	Thymus cell antigen 1, theta
Il2rb	Interleukin 2 receptor, beta chain
Cd28	CD28 antigen
H2-Aa	Histocompatibility 2, class II antigen A, alpha
Prom1	Prominin 1
H2-M2	Histocompatibility 2, M region locus 2
Cd8a	CD8 antigen, alpha chain
Fasl	Fas ligand (TNF superfamily, member 6)
Pdcd1	Programmed cell death 1
Klrc1	Killer cell lectin-like receptor subfamily C, member 1
Cd24a	CD24a antigen
Btla	B and T lymphocyte associated
Cd5	CD5 antigen
Ncam1	Neural cell adhesion molecule 1
Klrd1	Killer cell lectin-like receptor, subfamily D, member 1
Cd2	CD2 antigen
Cd34	CD34 antigen
Il17rb	Interleukin 17 receptor B

Cellular component—external side of plasma membrane—GO:0009897	
adj *P* = 1.43*e* − 06	
Cd8b1	CD8 antigen, beta chain 1
Cd5	CD5 antigen
Btla	B and T lymphocyte associated
Cd24a	CD24a antigen
Thy1	Thymus cell antigen 1, theta
Ncam1	Neural cell adhesion molecule 1
Il2rb	Interleukin 2 receptor, beta chain
Cd28	CD28 antigen
H2-Aa	Histocompatibility 2, class II antigen A, alpha
Cd2	CD2 antigen
Klrd1	Killer cell lectin-like receptor, subfamily D, member 1
Cd34	CD34 antigen
Cd8a	CD8 antigen, alpha chain
Fasl	Fas ligand (TNF superfamily, member 6)
Pdcd1	Programmed cell death 1
Klrc1	Killer cell lectin-like receptor subfamily C, member 1

Cellular component—plasma membrane part—GO:0044459	
adj *P* = 1.08*e* − 05	
Cd8b1	CD8 antigen, beta chain 1
Thy1	Thymus cell antigen 1, theta
Itgbl1	Integrin, beta-like 1
Cd28	CD28 antigen
Prom1	Prominin 1
Cd8a	CD8 antigen, alpha chain
Fasl	Fas ligand (TNF superfamily, member 6)
Slc39a5	Solute carrier family 39 (metal ion transporter), member 5
Tjp3	Tight junction protein 3
Klrc1	Killer cell lectin-like receptor subfamily C, member 1
Clec2h	C-type lectin domain family 2, member h
Gnat2	Guanine nucleotide binding protein, alpha transducing 2
Cd24a	CD24a antigen
Btla	B and T lymphocyte associated
Ncam1	Neural cell adhesion molecule 1
Igsf9	Immunoglobulin superfamily, member 9
Art2b	ADP-ribosyltransferase 2b
Klrd1	Killer cell lectin-like receptor, subfamily D, member 1
Dsg1b	Desmoglein 1 beta
Cd34	CD34 antigen
Muc3	Mucin 3, intestinal
Dsg1c	Desmoglein 1 gamma
Il17rb	Interleukin 17 receptor B
Rasgrp2	RAS, guanyl releasing protein 2
Slc26a3	Solute carrier family 26, member 3
Abcc3	ATP-binding cassette, sub-family C (CFTR/MRP), member 3
Lor	Loricrin
Cdh1	Cadherin 1
Il2rb	Interleukin 2 receptor, beta chain
Gpnmb	Glycoprotein (transmembrane) nmb
H2-Aa	Histocompatibility 2, class II antigen A, alpha
Espn	Espin
Cd3g	CD3 antigen, gamma polypeptide
Ms4a4b	Membrane-spanning 4-domains, subfamily A, member 4B
C8b	Complement component 8, beta polypeptide
Lat	Linker for activation of T cells
Grin3b	Glutamate receptor, ionotropic, NMDA3B
Aqp8	Aquaporin 8
Kcnn4	Potassium intermediate/small conductance calcium-activated channel, subfamily N, member 4
Slco1a1	Solute carrier organic anion transporter family, member 1a1
Pdcd1	Programmed cell death 1
Cd5	CD5 antigen
Grm8	Glutamate receptor, metabotropic 8
Cd2	CD2 antigen
Vsig2	V-set and immunoglobulin domain containing 2
Cd3d	CD3 antigen, delta polypeptide
Gna14	Guanine nucleotide binding protein, alpha 14

Cellular component—extracellular region part—GO:0044421	
adj *P* = 0.0043	
Col5a2	Collagen, type V, alpha 2
Vcan	Versican
Slc1a3	Solute carrier family 1 (glial high affinity glutamate transporter), member 3
Dmbt1	Deleted in malignant brain tumors 1
Ltb	Lymphotoxin B
Prom1	Prominin 1
Afp	Alpha fetoprotein
Calca	Calcitonin/calcitonin-related polypeptide, alpha
Sftpd	Surfactant associated protein D
Fasl	Fas ligand (TNF superfamily, member 6)
Orm3	Orosomucoid 3
Ccl5	Chemokine (C-C motif) ligand 5
Tff3	Trefoil factor 3, intestinal
Cpxm2	Carboxypeptidase X 2 (M14 family)
Cfd	Complement factor D (adipsin)
Ccl12	Chemokine (C-C motif) ligand 12
Ccl4	Chemokine (C-C motif) ligand 4
Crispld2	Cysteine-rich secretory protein LCCL domain containing 2
Adipoq	Adiponectin, C1Q and collagen domain containing
Mmp7	Matrix metallopeptidase 7
Ccl8	Chemokine (C-C motif) ligand 8
Gpc1	Glypican 1
Dpt	Dermatopontin
Cxcl9	Chemokine (C-X-C motif) ligand 9
Mmp8	Matrix metallopeptidase 8
Fam3b	Family with sequence similarity 3, member B

Cellular component—intrinsic to plasma membrane—GO:0031226	
adj *P* = 0.0093	
Slc26a3	Solute carrier family 26, member 3
Thy1	Thymus cell antigen 1, theta
Itgbl1	Integrin, beta-like 1
Abcc3	ATP-binding cassette, sub-family C (CFTR/MRP), member 3
Gpnmb	Glycoprotein (transmembrane) nmb
Ms4a4b	Membrane-spanning 4-domains, subfamily A, member 4B
Prom1	Prominin 1
C8b	Complement component 8, beta polypeptide
Lat	Linker for activation of T cells
Grin3b	Glutamate receptor, ionotropic, NMDA3B
Aqp8	Aquaporin 8
Kcnn4	Potassium intermediate/small conductance calcium-activated channel, subfamily N, member 4
Slco1a1	Solute carrier organic anion transporter family, member 1a1
Clec2h	C-type lectin domain family 2, member h
Cd24a	CD24a antigen
Btla	B and T lymphocyte associated
Grm8	Glutamate receptor, metabotropic 8
Cd2	CD2 antigen
Vsig2	V-set and immunoglobulin domain containing 2
Il17rb	Interleukin 17 receptor B
