# Supplementary material for: Hepatic Gene Expression Profiling in Nrf2 Knockout Mice after Long-Term High-Fat Diet-Induced Obesity
Source: Oxid Med Cell Longev. 2013 Apr 18;2013:340731. doi: 10.1155/2013/340731 (PMC3654373; doi:10.1155/2013/340731)
Supplement: Supplementary file 1 — The supplementary material comprises of two figures and three tables. A heatmap of the differentially expressed genes between WT and Nrf2-KO mice after HFD feeding is depicted in figure S1. Figure S2 shows a gene network of some of the differentially expressed genes that also comprises Nrf2. The primer sequences used in this study are provided in Table S1. The 428 genes that were over-expressed as well the 173 genes that were under-expressed in the liver of the Nrf2-KO mice compared to their WT counterparts after 180 days on HFD, are presented in Tables S2 and S3 correspondingly. [file 340731.f1.pdf]

SUPPLEMENTARY FIGURES

FIG.S1

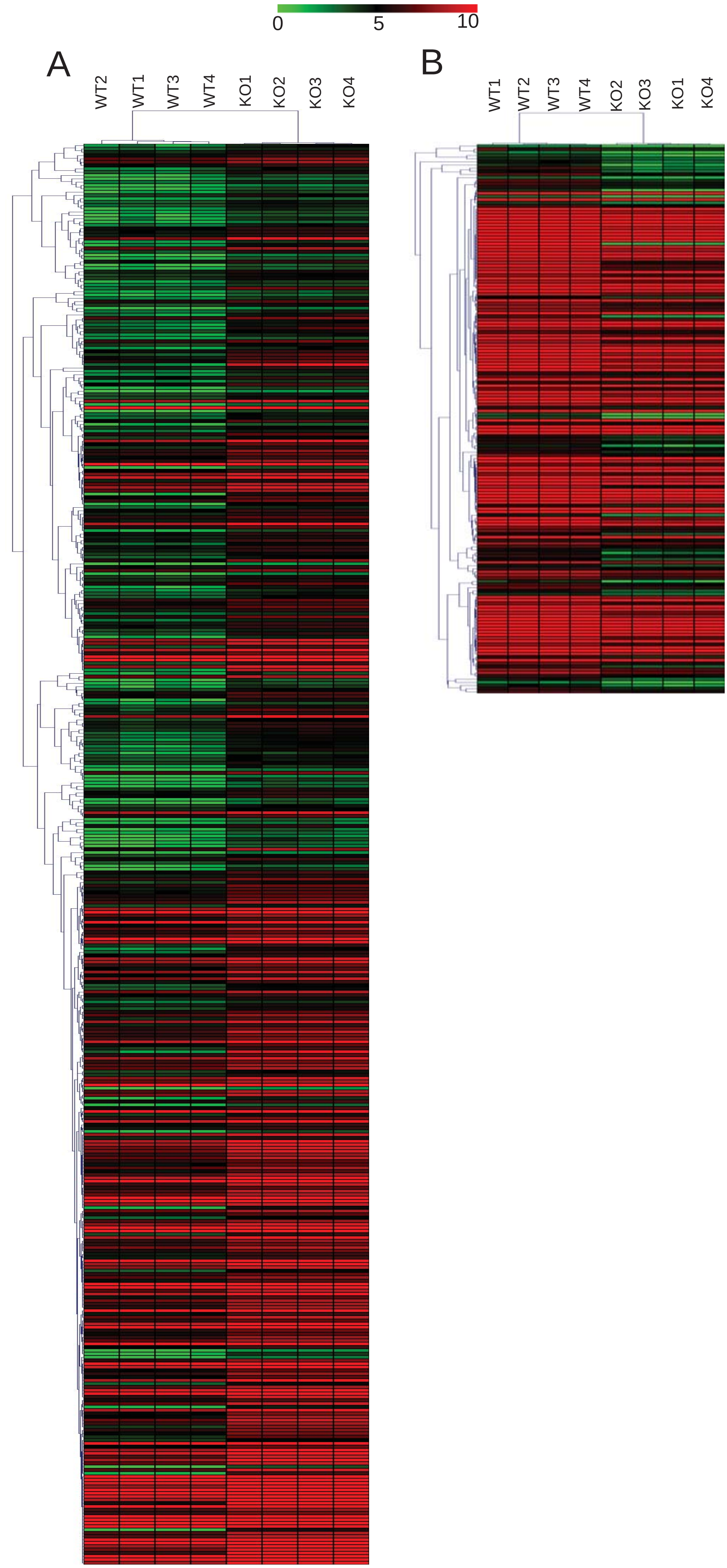
0
5
10
A
WT2
WT1
WT3
WT4
KO1
KO2
KO3
KO4
B
WT1
WT2
WT3
WT4
KO2
KO3
KO1
KO4

FIG.S2

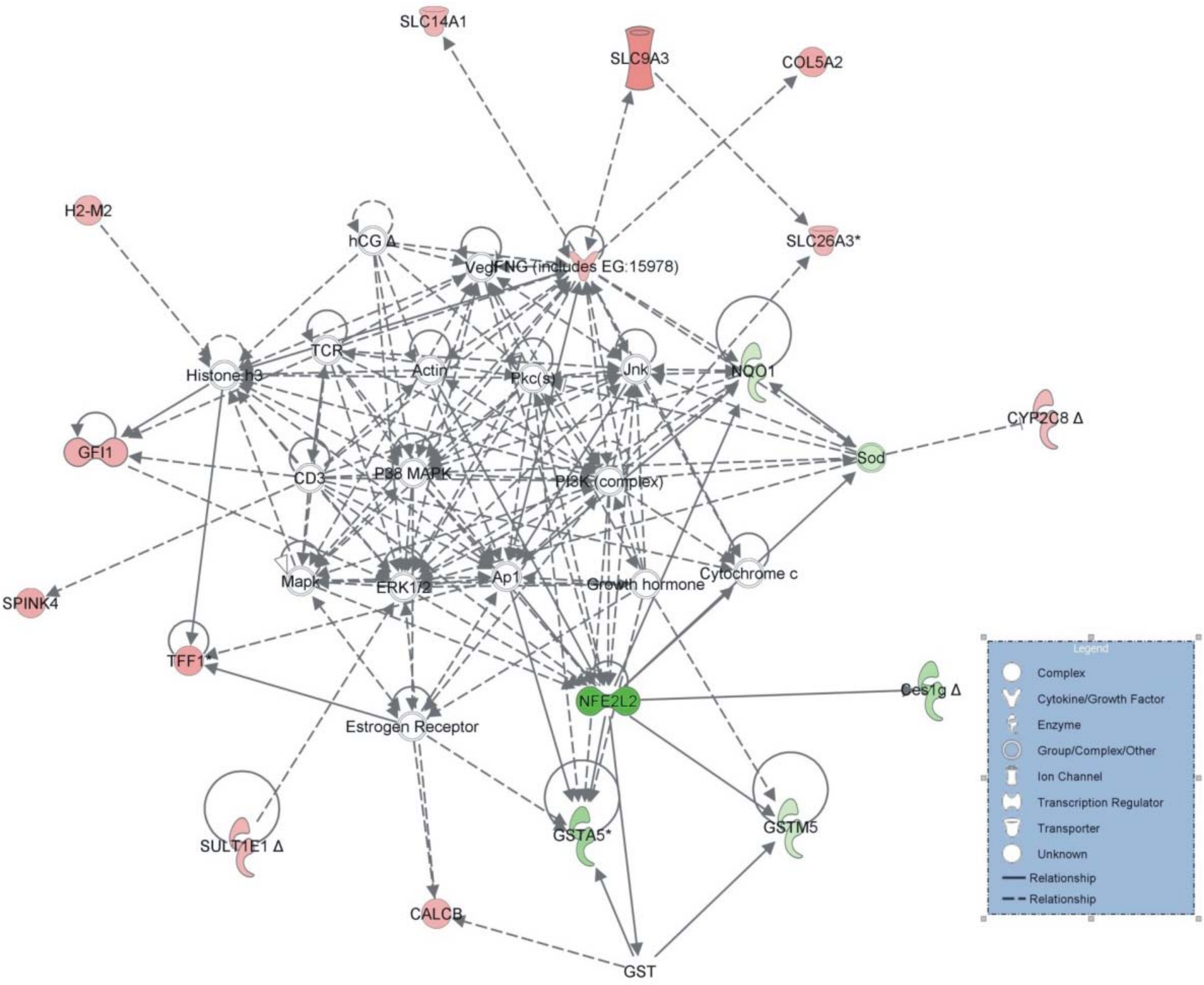

SLC14A1
SLC9A3
COL5A2
H2-M2
hCG Δ
Vegf
IFNG (includes EG:15978)
SLC26A3*
Histone h3
TCR
Actin
Pkc(s)
Jnk
NQO1
CYP2C8 Δ
GFI1
CD3
P38 MAPK
PI3K (complex)
Sod
Mapk
ERK1/2
Ap1
Growth hormone
Cytochrome c
SPINK4
TFF1*
NFE2L2
Ces1g Δ
Estrogen Receptor
SULT1E1 Δ
GSTA5*
GSTM5
CALCB
GST
Legend
Complex
Cytokine/Growth Factor
Enzyme
Group/Complex/Other
Ion Channel
Transcription Regulator
Transporter
Unknown
Relationship
Relationship

SUPPLEMENTARY TABLES

Table S1. Primer sequences used in quantitative RT-PCR.

Gene	Forward Primer	Reverse Primer	PrimerBank ID
Cyp7a1	GCTGTGGTAGTGAGCTGTTG	AGGAGGTTCACCTACTTTCCTT	31542444b1
Fabp5	AAAGAGCTAGGAGTAGGACTGG	TGTTGCCATCACACGTAATGA	118130073b1
Me1	ACAAAGGGCACATTGCTTCAG	TTACCACAATAGCCTTGACGAC	162139826b3
Lipocalin 13	TCATAAAGCGCATGAATCTCCG	ATCTCCGATCTCTGGCACAAA	23956341b2
Aquaporin 8	CATCGGATGTCTATCGGTCATTG	AGGAGCCCAGTATTCGGACTA	157824035b2
Cyp2b10	TTGTTACTCTTAGCCAGGGGAC	GGAAGTTGCCACGGGACTTT	118129803b1
Car	CCCTGACAGACCCGGAGTTA	GCCGAGACTGTTGTTCCATAAT	6754888a1
Cbr3	CGGGCATCGCCTTTAGAATG	GCTTGAATGTCGAAGGGTGTT	226823287b2
Nqo1	GGCATCCTGCGTTTCTGTG	GGTTTCCAGACGTTTCTTCCAT	6679080A3

Cyp7a1 (cytochrome P450, family 7, subfamily A, polypeptide 1), *Fabp5* (fatty acid binding protein 5), *Me1* (malic enzyme 1),*Cyp2b10* (cytochrome P450, family 2, subfamily B, polypeptide 10), *Car* (constitutive androstane receptor), , *Cbr3* (carbonyl reductase 3), , *Nqo1* (NAD(P)H dehydrogenase, quinone 1).

Table S2. 428 genes that were over-expressed in the liver of the Nrf2 knock-out mice compared to the wild-type mice after 180 days on high-fat diet.

Probe ID	Primary Accession	Gene Symbol	Gene Name	fold change
A_55_P2144686	NM_007769	Dmbt1	deleted in malignant brain tumors 1	138.3
A_55_P1982499	NM_177350	Gldn	gliomedin	124.6
A_51_P493987	NM_021509	Moxd1	"monooxygenase, DBH-like 1"	86.3
A_51_P329811	NM_009042	Reg1	regenerating islet-derived 1	74.95
A_51_P456208	NM_011575	Tff3	"trefoil factor 3, intestinal"	84.32
A_51_P450406	AK132919	Muc3	"mucin 3, intestinal"	29.47
A_52_P250400	NM_178685	Pcdh20	protocadherin 20	19.82
A_51_P434758	NM_001033170	Fam83e	"family with sequence similarity 83, member E"	19.48
A_55_P2362635	AK033564	Slc9a3	"solute carrier family 9 (sodium/hydrogen exchanger), member 3"	15.85
A_55_P2075253	XM_001475531	Gm3054	predicted gene 3054	17.15
A_55_P2047163	NM_001105252	Tmc5	transmembrane channel-like gene family 5	12.34
A_52_P566840	NM_133776	Gpr110	G protein-coupled receptor 110	13.8
A_55_P2025687	NM_080457	Muc4	mucin 4	9.471
A_52_P429450	NM_008694	Ngp	neutrophilic granule protein	9.493
A_55_P2048855	NM_001164787	Sprr2a2	small proline-rich protein 2A2	13.15
A_51_P285736	NM_008798	Pdcd1	programmed cell death 1	9.304
A_55_P2089076	NM_001099314	Msmp	"microseminoprotein, prostate associated"	10.25
A_55_P2078281	AY324401	NA	NA	9.404
A_55_P2042331	ENSMUST00000122399	NA	NA	8.83
A_55_P2112005	NM_009362	Tff1	trefoil factor 1	6.234
A_55_P2137049	NM_001004174	AA467197	expressed sequence AA467197	9.65
A_52_P154101	NM_001033954	Calca	"calcitonin/calcitonin-related polypeptide, alpha"	6.316
A_51_P464703	NM_021443	Ccl8	chemokine (C-C motif) ligand 8	7.859
A_55_P2083014	U63547	NA	NA	8.295
A_55_P1956147	NM_008081	B4galnt2	"beta-1,4-N-acetyl-galactosaminyl transferase 2"	7.044
A_55_P2162695	X03802	NA	NA	5.262
A_52_P650453	NM_011463	Spink4	"serine peptidase inhibitor, Kazal type 4"	7.415
A_55_P2083963	NM_001145644	Phgr1	proline/histidine/glycine-rich 1	7.38
A_51_P419226	NM_025393	S100a14	S100 calcium binding protein A14	8.124
A_55_P2186942	ENSMUST00000103330	NA	NA	7.192
A_55_P2145389	NM_001172160	Flrt3	fibronectin leucine rich transmembrane protein 3	7.404
A_55_P1977008	NM_010278	Gfi1	growth factor independent 1	6.642
A_55_P2025586	ENSMUST00000103483	NA	NA	6.491
A_55_P2220748	NM_008586	Mep1b	meprin 1 beta	5.911
A_52_P31381	NM_021353	Slc26a3	"solute carrier family 26, member 3"	5.525

Table S2. (continued)

A_55_P2157384	NM_001111293	Xlr5b	X-linked lymphocyte-regulated 5B	6.206
A_55_P2104894	NM_007737	Col5a2	"collagen, type V, alpha 2"	6.913
A_52_P307904	NM_172825	Gpr128	G protein-coupled receptor 128	5.256
A_55_P2103948	ENSMUST00000103361	NA	NA	7.246
A_55_P2161794	NM_028191	Cyp2c65	"cytochrome P450, family 2, subfamily c, polypeptide 65"	4.89
A_52_P279425	NM_032465	Cd96	CD96 antigen	5.815
A_55_P2135064	NM_008204	H2-M2	"histocompatibility 2, M region locus 2"	5.596
A_52_P447284	NM_172469	Clic6	chloride intracellular channel 6	7.266
A_51_P312336	NM_028122	Slc14a1	"solute carrier family 14 (urea transporter), member 1"	5.615
A_55_P1959421	ENSMUST00000103379	NA	NA	6.033
A_51_P493649	NM_023135	Sult1e1	"sulfotransferase family 1E, member 1"	5.486
A_55_P1969087	ENSMUST00000053475	NA	NA	3.869
A_55_P2110925	NM_001172160	Flrt3	fibronectin leucine rich transmembrane protein 3	5.459
A_52_P15388	NM_008522	Ltf	lactotransferrin	5.331
A_55_P2073024	NM_001034859	Gm4841	predicted gene 4841	5.58
A_55_P1995537	NM_010824	Mpo	myeloperoxidase	5.502
A_55_P1958146	NM_011364	Sh2d1a	SH2 domain protein 1A	5.991
A_55_P2092219	NM_027997	Serpina9	"serine (or cysteine) peptidase inhibitor, clade A (alpha-1 antiproteinase, antit	5.38
A_55_P1954231	NM_172492	Lrtm2	leucine-rich repeats and transmembrane domains 2	5.684
A_55_P2115981	NM_031493	Xlr5c	X-linked lymphocyte-regulated 5C	5.546
A_55_P2058028	ENSMUST00000103351	NA	NA	5.472
A_55_P2034655	AK007918	NA	NA	5.243
A_52_P312102	ENSMUST00000090180	Sema3g	"sema domain, immunoglobulin domain (Ig), short basic domain, secreted, (s	5.709
A_66_P114826	ENSMUST00000103336	NA	NA	4.996
A_51_P509679	BC004786	Igh-VJ558	immunoglobulin heavy chain (J558 family)	5.132
A_52_P480044	XM_001471580	BC023105	cDNA sequence BC023105	4.9
A_51_P426096	NM_010810	Mmp7	matrix metallopeptidase 7	4.844
A_51_P356642	NM_008471	Krt19	keratin 19	4.925
A_52_P200125	M34969	NA	NA	4.41
A_55_P2153762	XM_916728	LOC640020	similar to Probable G-protein coupled receptor 110 precursor (G-protein coup	4.891
A_55_P1971872	XM_001477072	LOC100046877	similar to developmentally regulated RNA-binding protein 1	5.259
A_55_P2111322	ENSMUST00000103741	NA	NA	4.878
A_55_P1956598	BC019489	NA	NA	4.816
A_55_P2144556	NM_001172160	Flrt3	fibronectin leucine rich transmembrane protein 3	4.795
A_55_P1984362	M11860	NA	NA	4.344
A_55_P2111980	NM_008295	Hsd3b5	"hydroxy-delta-5-steroid dehydrogenase, 3 beta- and steroid delta-isomerase	4.697

Table S2. (continued)

A_51_P115601	ENSMUST00000103364	NA	NA	5.418
A_55_P1960148	AK007826	NA	NA	4.613
A_55_P2063283	AK135975	Ighg	Immunoglobulin heavy chain (gamma polypeptide)	4.657
A_55_P2066173	AK041235	NA	NA	4.823
A_55_P2158102	NM_009921	Camp	cathelicidin antimicrobial peptide	4.816
A_55_P2164977	XM_001473158	Gm2330	predicted gene 2330	5.007
A_55_P2062246	NM_001145164	Tgtp2	T-cell specific GTPase 2	4.492
A_55_P2016114	NM_010177	Fasl	"Fas ligand (TNF superfamily, member 6)"	4.924
A_55_P2047589	ENSMUST00000103328	NA	NA	4.407
A_55_P2020148	NM_001001792	Zfp239	zinc finger protein 239	4.601
A_55_P2133907	ENSMUST00000103520	NA	NA	4.939
A_52_P638459	NM_013653	Ccl5	chemokine (C-C motif) ligand 5	4.395
A_52_P1190151	ENSMUST00000103328	NA	NA	5.177
A_55_P2177243	NM_145920	Evc2	Ellis van Creveld syndrome 2 homolog (human)	4.075
A_55_P2005984	NM_138685	Wfdc15b	WAP four-disulfide core domain 15B	4.446
A_51_P492047	NM_020622	Fam3b	"family with sequence similarity 3, member B"	3.911
A_55_P1987406	ENSMUST00000103746	NA	NA	4.538
A_51_P406253	NM_008100	Gcg	glucagon	3.934
A_51_P212068	NM_029638	Abp1	"amiloride binding protein 1 (amine oxidase, copper-containing)"	4.229
A_51_P270904	NM_172641	9930023K05Rik	RIKEN cDNA 9930023K05 gene	4.611
A_51_P499195	NM_024253	Nkg7	natural killer cell group 7 sequence	4.221
A_51_P259029	NM_025869	Dusp26	dual specificity phosphatase 26 (putative)	4.483
A_55_P1961127	NR_001592	H19	H19 fetal liver mRNA	4.133
A_66_P113466	ENSMUST00000103334	NA	NA	4.083
A_55_P2316041	BC050972	BC050972	cDNA sequence BC050972	3.799
A_55_P2072771	NM_009850	Cd3g	"CD3 antigen, gamma polypeptide"	4.107
A_55_P2150717	NM_010136	Eomes	eomesodermin homolog (Xenopus laevis)	4.297
A_55_P2214124	BC019425	Igh-VJ558	immunoglobulin heavy chain (J558 family)	3.963
A_55_P2026238	NA	NA	NA	4.616
A_55_P2165199	NM_030712	Cxcr6	chemokine (C-X-C motif) receptor 6	4.087
A_55_P2011341	XM_001474162	LOC100045268	similar to precursor (AA -28 to 232)	3.957
A_55_P1987151	NM_011860	Nlrp5	"NLR family, pyrin domain containing 5"	4.093
A_55_P2039699	NM_009921	Camp	cathelicidin antimicrobial peptide	4.248
A_52_P112591	U07662	NA	NA	4.779
A_55_P1962209	NM_030712	Cxcr6	chemokine (C-X-C motif) receptor 6	3.938
A_66_P140474	ENSMUST00000103348	LOC100047316	similar to anti-MOG Z12 variable light chain	3.931

Table S2. (continued)

A_52_P484956	NM_010923	Nnat	neuronatin	3.895
A_52_P374897	NM_009705	Arg2	arginase type II	4.273
A_52_P52849	NM_018867	Cpxm2	carboxypeptidase X 2 (M14 family)	3.827
A_51_P367866	NM_007913	Egr1	early growth response 1	4.048
A_55_P2045136	AK153212	I830127L07Rik	RIKEN cDNA I830127L07 gene	3.533
A_55_P2020428	NM_146232	BC014805	cDNA sequence BC014805	3.745
A_55_P2161347	NM_001033041	Acmsd	amino carboxymuconate semialdehyde decarboxylase	3.861
A_51_P507290	NM_009769	Klf5	Kruppel-like factor 5	3.937
A_55_P2249556	AK042310	A630081D01Rik	RIKEN cDNA A630081D01 gene	3.757
A_51_P150710	NM_152839	Igj	immunoglobulin joining chain	3.793
A_55_P2058023	ENSMUST00000103356	NA	NA	3.999
A_52_P374653	ENSMUST00000103397	NA	NA	3.851
A_55_P2206605	AK030886	5830444B04Rik	RIKEN cDNA 5830444B04 gene	3.97
A_51_P448147	NM_146167	Gimap7	"GTPase, IMAP family member 7"	3.831
A_51_P458451	NM_009605	Adipoq	"adiponectin, C1Q and collagen domain containing"	3.589
A_55_P2094060	NM_010370	Gzma	granzyme A	3.698
A_55_P2004761	NM_001033978	H2-Eb2	"histocompatibility 2, class II antigen E beta2"	3.524
A_52_P112190	ENSMUST00000103377	LOC100047053	similar to monoclonal antibody kappa light chain	3.732
A_55_P2186978	ENSMUST00000103352	NA	NA	3.86
A_55_P2092750	NM_139305	Car9	carbonic anhydrase 9	3.666
A_66_P102346	ENSMUST00000103321	NA	NA	3.472
A_55_P2121701	AK140370	E530001K10Rik	RIKEN cDNA E530001K10 gene	3.634
A_51_P485756	NM_024435	Nts	neurotensin	3.598
A_55_P2046653	ENSMUST00000103460	LOC641221	similar to Ig heavy chain V region PJ14 precursor	3.544
A_51_P269404	NM_008030	Fmo3	flavin containing monooxygenase 3	3.614
A_55_P2013840	NM_212483	Krt42	keratin 42	3.986
A_52_P502849	ENSMUST00000103401	NA	NA	3.661
A_66_P139675	M11858	NA	NA	3.506
A_51_P137419	NM_009977	Cst7	cystatin F (leukocystatin)	3.568
A_55_P2002744	ENSMUST00000103528	NA	NA	3.626
A_55_P2058040	ENSMUST00000103345	NA	NA	3.828
A_51_P478172	AY324401	NA	NA	3.598
A_51_P136781	NM_018874	Pnliprp1	pancreatic lipase related protein 1	3.558
A_51_P122649	NM_027299	Degs2	"degenerative spermatocyte homolog 2 (Drosophila), lipid desaturase"	3.521
A_52_P443334	NM_001081110	Cd8a	"CD8 antigen, alpha chain"	3.652
A_55_P1977038	NM_008599	Cxcl9	chemokine (C-X-C motif) ligand 9	3.704

Table S2. (continued)

A_66_P105596	ENSMUST00000103396	LOC676193	similar to Ig kappa chain V-III region PC 6684	3.743
A_55_P2094104	ENSMUST00000103380	LOC100047070	"similar to [Human Ig rearranged gamma chain mRNA, V-J-C region and com	3.785
A_66_P109692	ENSMUST00000103332	NA	NA	3.501
A_55_P2183369	ENSMUST00000103401	NA	NA	3.862
A_66_P106760	NM_153397	Adam32	a disintegrin and metallopeptidase domain 32	3.513
A_51_P174696	NM_009814	Casq2	calsequestrin 2	3.227
A_65_P07626	ENSMUST00000103403	NA	NA	3.602
A_51_P167292	NM_009892	Chi3l3	chitinase 3-like 3	3.435
A_51_P363400	NM_008920	Prg2	"proteoglycan 2, bone marrow"	3.643
A_55_P2003911	ENSMUST00000103391	NA	NA	3.639
A_55_P2065506	ENSMUST00000103513	LOC639930	similar to Ig heavy chain V-I region HG3 precursor	3.829
A_51_P136294	NM_021718	Ms4a4b	"membrane-spanning 4-domains, subfamily A, member 4B"	3.566
A_52_P614207	ENSMUST00000103399	LOC100047222	similar to anti-glycoprotein-B of human Cytomegalovirus immunoglobulin Vl c	3.4
A_55_P2153517	NM_027147	Enho	energy homeostasis associated	3.429
A_55_P2015753	NM_027147	Enho	energy homeostasis associated	3.519
A_55_P2156653	NM_001177781	Gpr174	G protein-coupled receptor 174	4.176
A_51_P285206	NM_013487	Cd3d	"CD3 antigen, delta polypeptide"	3.419
A_51_P461665	NM_008599	Cxcl9	chemokine (C-X-C motif) ligand 9	3.383
A_55_P2390776	NR_027981	1810064F22Rik	RIKEN cDNA 1810064F22 gene	3.377
A_55_P1978033	ENSMUST00000103397	NA	NA	3.535
A_51_P268697	NM_148938	Slc1a3	"solute carrier family 1 (glial high affinity glutamate transporter), member 3"	3.285
A_55_P1996702	NM_008174	Grm8	"glutamate receptor, metabotropic 8"	3.382
A_55_P2065510	ENSMUST00000103506	NA	NA	3.451
A_55_P2416494	AK020235	8430426J06Rik	RIKEN cDNA 8430426J06 gene	3.479
A_55_P1998471	NM_009114	S100a9	S100 calcium binding protein A9 (calgranulin B)	3.359
A_51_P395899	ENSMUST00000103404	LOC100047628	"similar to Chain L, Structural Basis Of Antigen Mimicry In A Clinically Releva	3.34
A_55_P2140348	BC103785	Igh-VJ558	immunoglobulin heavy chain (J558 family)	3.206
A_51_P256827	NM_013650	S100a8	S100 calcium binding protein A8 (calgranulin A)	3.404
A_55_P1956083	NM_175493	Gpr68	G protein-coupled receptor 68	3.48
A_55_P2026903	NM_001146325	Tigit	T cell immunoreceptor with Ig and ITIM domains	3.075
A_55_P2154982	NM_009858	Cd8b1	"CD8 antigen, beta chain 1"	3.199
A_55_P1966608	XM_917762	LOC640701	similar to Ig heavy chain V-I region V35 precursor	3.623
A_51_P478722	NM_011579	Tgtp1	T-cell specific GTPase 1	3.146
A_55_P1970464	ENSMUST00000103507	NA	NA	3.306
A_55_P2060897	ENSMUST00000103410	NA	NA	3.238
A_51_P497741	NM_029440	4930434E21Rik	RIKEN cDNA 4930434E21 gene	3.236

Table S2. (continued)

A_52_P563375	NM_025622	Lgals2	"lectin, galactose-binding, soluble 2"	3.182
A_52_P195135	NM_133353	Oosp1	oocyte secreted protein 1	3.063
A_55_P2007601	NM_009160	Sftpd	surfactant associated protein D	3.31
A_51_P493016	NM_023821	Cmya5	cardiomyopathy associated 5	2.749
A_55_P2001262	ENSMUST00000109711	NA	NA	3.385
A_51_P405638	ENSMUST00000103473	LOC100045791	similar to monoclonal antibody heavy chain	3.049
A_55_P2012592	NM_134109	Ildr1	immunoglobulin-like domain containing receptor 1	2.786
A_55_P1977451	ENSMUST00000103341	NA	NA	3.43
A_55_P2138627	ENSMUST00000103486	LOC675759	similar to Ig heavy chain V-III region J606	3.525
A_66_P127563	ENSMUST00000103360	NA	NA	3.223
A_66_P106497	ENSMUST00000103378	NA	NA	3.174
A_52_P618728	ENSMUST00000103362	NA	NA	3.117
A_55_P1984556	NM_011331	Ccl12	chemokine (C-C motif) ligand 12	3.116
A_52_P578436	ENSMUST00000103347	NA	NA	3.222
A_55_P1969901	ENSMUST00000103547	NA	NA	3.404
A_51_P171772	NM_021399	Bcl11b	B-cell leukemia/lymphoma 11B	3.234
A_55_P2005838	NM_029835	5730590G19Rik	RIKEN cDNA 5730590G19 gene	3.079
A_55_P1964886	NM_019915	Art2b	ADP-ribosyltransferase 2b	3.033
A_51_P513776	ENSMUST00000103535	LOC640979	similar to Ig heavy chain V region 23 precursor	3.138
A_51_P419656	NM_020268	Klk1b27	kallikrein 1-related peptidase b27	2.882
A_51_P288295	ENSMUST00000103496	LOC677484	similar to Ig heavy chain V region B1-8/186-2 precursor	3.13
A_55_P2059010	NM_011254	Rbp1	"retinol binding protein 1, cellular"	3.122
A_55_P2135986	NM_029499	Ms4a4c	"membrane-spanning 4-domains, subfamily A, member 4C"	3.105
A_66_P109519	NM_007914	Ehf	ets homologous factor	3.039
A_55_P1983921	NM_021352	Crybb3	"crystallin, beta B3"	3.122
A_52_P320193	NM_053165	Clec2h	"C-type lectin domain family 2, member h"	3.005
A_55_P2107374	NM_025777	Duoxa2	dual oxidase maturation factor 2	3.051
A_55_P2001281	ENSMUST00000103523	NA	NA	3.377
A_55_P2096797	NM_001040696	Nlrp1b	"NLR family, pyrin domain containing 1B"	3.416
A_55_P1988975	BC028433	Ms4a4b	"membrane-spanning 4-domains, subfamily A, member 4B"	3.002
A_66_P108380	NM_172447	A330021E22Rik	RIKEN cDNA A330021E22 gene	3.04
A_55_P1989225	NM_001145164	Tgtp2	T-cell specific GTPase 2	2.907
A_55_P2063880	ENSMUST00000110689	NA	NA	2.947
A_55_P2019989	XM_001479535	Gm4249	predicted gene 4249	2.863
A_55_P2011950	ENSMUST00000103552	LOC435333	similar to monoclonal antibody heavy chain	2.977
A_51_P221510	NM_029784	Fam81a	"family with sequence similarity 81, member A"	2.845

Table S2. (continued)

A_51_P203955	NM_010260	Gbp2	guanylate binding protein 2	2.904
A_55_P2001269	ENSMUST00000103522	NA	NA	2.896
A_52_P184629	M25913	NA	NA	2.523
A_55_P1996973	NM_029000	Gvin1	"GTPase, very large interferon inducible 1"	2.895
A_55_P2105454	ENSMUST00000103458	NA	NA	3.155
A_55_P2021275	NM_029122	Iqca	IQ motif containing with AAA domain	2.739
A_51_P487073	NM_138684	Wfdc12	WAP four-disulfide core domain 12	2.8
A_55_P2030299	ENSMUST00000103469	LOC544904	similar to Ig heavy chain V region 186-1 precursor	2.869
A_55_P2100194	BC100303	A530040E14Rik	RIKEN cDNA A530040E14 gene	2.912
A_51_P140237	NM_010212	Fhl2	four and a half LIM domains 2	2.996
A_52_P142191	NM_177583	Aph1b	anterior pharynx defective 1b homolog (C. elegans)	2.89
A_55_P2124586	NM_001081642	Xlr4a	X-linked lymphocyte-regulated 4A	2.981
A_51_P485421	ENSMUST00000103426	NA	NA	2.969
A_55_P2039230	XM_001477580	LOC100047175	hypothetical protein LOC100047175	2.702
A_52_P213483	AK007918	NA	NA	3.154
A_52_P501875	NM_001077684	4930578N16Rik	RIKEN cDNA 4930578N16 gene	3.264
A_51_P455997	NR_027652	Meg3	maternally expressed 3	2.581
A_52_P150547	NM_009857	Cd8a	"CD8 antigen, alpha chain"	2.852
A_51_P509573	NM_013652	Ccl4	chemokine (C-C motif) ligand 4	2.801
A_55_P2087429	ENSMUST00000103393	LOC100047162	similar to immunoglobulin kappa-chain	2.789
A_55_P1988844	NM_016970	Klrg1	"killer cell lectin-like receptor subfamily G, member 1"	2.678
A_55_P2068673	NM_025285	Stmn2	stathmin-like 2	2.896
A_55_P2186972	ENSMUST00000103342	NA	NA	3.163
A_55_P2117081	M54990	NA	NA	2.656
A_51_P389636	NM_008433	Kcnn4	"potassium intermediate/small conductance calcium-activated channel, subfa	2.654
A_55_P1976450	XM_001004859	NA	NA	2.963
A_55_P2103837	NM_008620	Gbp4	guanylate binding protein 4	2.596
A_51_P116601	NM_172447	A330021E22Rik	RIKEN cDNA A330021E22 gene	2.725
A_55_P2042076	NM_145592	Dkk4	dickkopf homolog 4 (Xenopus laevis)	2.672
A_51_P503896	BC006727	Pycr1	pyrroline-5-carboxylate reductase 1	2.539
A_55_P2145711	NM_172913	Tox3	TOX high mobility group box family member 3	2.74
A_52_P343627	NM_022020	Rbp7	"retinol binding protein 7, cellular"	2.596
A_55_P2108077	M13677	NA	NA	2.484
A_55_P2001267	XM_001472404	NA	NA	4.073
A_55_P1999902	AK167816	Pip5k1a	"phosphatidylinositol-4-phosphate 5-kinase, type 1 alpha"	2.742
A_55_P2169356	AK156879	Gm1966	predicted gene 1966	2.696

Table S2. (continued)

A_55_P2179599	NM_029509	Gbp8	guanylate-binding protein 8	2.639
A_51_P426270	NM_008597	Mgp	matrix Gla protein	2.68
A_55_P2115906	NM_181680	Dsg1c	desmoglein 1 gamma	2.549
A_55_P1999715	M97158	NA	NA	2.635
A_51_P473498	NM_173398	Gpr171	G protein-coupled receptor 171	2.569
A_55_P2171196	XM_001476596	Gm3411	predicted gene 3411	2.565
A_52_P295104	NM_183259	2210020M01Rik	RIKEN cDNA 2210020M01 gene	2.638
A_51_P435922	NM_029338	Rsph9	radial spoke head 9 homolog (Chlamydomonas)	2.62
A_51_P350817	NM_009922	Cnn1	calponin 1	2.601
A_55_P2176753	XM_917662	LOC640614	similar to Ig heavy chain V region 93G7 precursor	2.555
A_51_P517001	NM_172491	D130040H23Rik	RIKEN cDNA D130040H23 gene	1.91
A_55_P1960202	NM_001081249	Vcan	versican	2.371
A_51_P398766	NM_010259	Gbp1	guanylate binding protein 1	2.474
A_55_P2130497	ENSMUST00000103538	NA	NA	2.604
A_55_P2121294	NA	NA	NA	2.267
A_52_P488437	NM_013738	Plek2	pleckstrin 2	2.537
A_55_P2146214	NM_001035510	Zcchc18	"zinc finger, CCHC domain containing 18"	2.536
A_51_P167263	NM_007650	Cd5	CD5 antigen	2.573
A_51_P322972	NM_145419	Hkdc1	hexokinase domain containing 1	2.513
A_51_P104768	ENSMUST00000103498	Gm16697	"predicted gene, 16697"	2.695
A_66_P124179	NM_175406	Atp6v0d2	"ATPase, H+ transporting, lysosomal V0 subunit D2"	2.472
A_55_P2059931	NM_001163577	Prom1	prominin 1	2.535
A_55_P1953387	NM_010634	Fabp5	"fatty acid binding protein 5, epidermal"	2.506
A_52_P431615	XM_001000891	Gm1966	predicted gene 1966	2.47
A_55_P1961715	NM_080637	Nme5	"non-metastatic cells 5, protein expressed in (nucleoside-diphosphate kinase	2.485
A_52_P535484	NM_029000	Gvin1	"GTPase, very large interferon inducible 1"	2.496
A_66_P106388	NM_029499	Ms4a4c	"membrane-spanning 4-domains, subfamily A, member 4C"	2.485
A_55_P2187067	ENSMUST00000103317	NA	NA	2.476
A_52_P30641	ENSMUST00000103337	NA	NA	2.796
A_55_P1952768	XM_001481046	LOC100048720	similar to guanylate nucleotide binding protein 1	2.477
A_52_P266132	NM_008013	Fgl2	fibrinogen-like protein 2	2.517
A_55_P1958464	NAP112102-1	NA	NA	2.458
A_51_P301930	NM_028977	Lrrc17	leucine rich repeat containing 17	2.527
A_52_P259817	NM_029692	Upp2	uridine phosphorylase 2	2.515
A_55_P2050439	NM_144553	Dlgap5	"discs, large (Drosophila) homolog-associated protein 5"	2.638
A_55_P1979364	ENSMUST00000103550	NA	NA	2.591

Table S2. (continued)

A_55_P2011794	XM_911832	LOC636441	similar to Ig heavy chain V region VH558 A1/A4 precursor	2.62
A_55_P2026547	NM_199366	Gal3st2	galactose-3-O-sulfotransferase 2	2.501
A_55_P2003915	ENSMUST00000103386	NA	NA	2.395
A_55_P2168823	NAP111644-1	NA	NA	2.407
A_51_P150120	NM_174995	Mgst2	microsomal glutathione S-transferase 2	2.376
A_55_P2097219	NM_010259	Gbp1	guanylate binding protein 1	2.512
A_51_P461067	AK041235	NA	NA	2.43
A_52_P149577	NM_016899	Rab25	"RAB25, member RAS oncogene family"	2.291
A_55_P2046101	NM_021365	Xlr4b	X-linked lymphocyte-regulated 4B	2.453
A_55_P2115900	NM_181682	Dsg1b	desmoglein 1 beta	2.473
A_51_P365516	NM_009258	Spink3	"serine peptidase inhibitor, Kazal type 3"	2.439
A_55_P2187030	ENSMUST00000103537	NA	NA	2.486
A_55_P1969665	NM_001111336	Hsd3b4	"hydroxy-delta-5-steroid dehydrogenase, 3 beta- and steroid delta-isomerase	2.062
A_66_P133148	NM_012054	Aoah	acyloxyacyl hydrolase	2.398
A_52_P615247	AK087744	A530040E14Rik	RIKEN cDNA A530040E14 gene	2.415
A_51_P231320	NM_008611	Mmp8	matrix metallopeptidase 8	2.371
A_55_P2042813	NM_001039647	Gbp11	guanylate binding protein 11	2.439
A_51_P286496	NM_008368	Il2rb	"interleukin 2 receptor, beta chain"	2.399
A_55_P1998943	NM_145211	Oas1a	2'-5' oligoadenylate synthetase 1A	2.435
A_55_P2101696	NM_008141	Gnat2	"guanine nucleotide binding protein, alpha transducing 2"	2.35
A_55_P2180176	NM_027209	Ms4a6b	"membrane-spanning 4-domains, subfamily A, member 6B"	2.418
A_55_P2124582	NM_001081642	Xlr4a	X-linked lymphocyte-regulated 4A	2.433
A_55_P2019690	NM_001081349	Slc43a1	"solute carrier family 43, member 1"	2.379
A_55_P2122841	NAP114472-1	NA	NA	2.385
A_52_P456750	NM_177583	Aph1b	anterior pharynx defective 1b homolog (C. elegans)	2.43
A_55_P2052385	NM_194336	Mpa2l	macrophage activation 2 like	2.369
A_55_P1968723	NM_009834	Ccrn4l	CCR4 carbon catabolite repression 4-like (S. cerevisiae)	2.365
A_52_P441974	NM_145920	Evc2	Ellis van Creveld syndrome 2 homolog (human)	2.36
A_51_P177092	NM_011487	Stat4	signal transducer and activator of transcription 4	2.387
A_55_P1983448	NM_011311	S100a4	S100 calcium binding protein A4	2.351
A_55_P2155504	NM_010693	Lck	lymphocyte protein tyrosine kinase	2.353
A_66_P119518	NM_017379	Tuba8	"tubulin, alpha 8"	2.431
A_51_P438967	NM_053110	Gpnmb	glycoprotein (transmembrane) nmb	2.447
A_55_P2150976	ENSMUST00000072582	Fabp5l2	fatty acid binding protein 5-like 2	2.344
A_66_P121053	NM_009160	Sftpd	surfactant associated protein D	2.304
A_55_P2051159	NM_029692	Upp2	uridine phosphorylase 2	2.385

Table S2. (continued)

A_51_P495242	NM_010689	Lat	linker for activation of T cells	2.569
A_51_P283344	NM_025956	1700011H14Rik	RIKEN cDNA 1700011H14 gene	2.359
A_55_P2077901	NM_013486	Cd2	CD2 antigen	2.338
A_51_P390937	ENSMUST00000103320	LOC675735	similar to Ig kappa chain V-V region L6 precursor	2.335
A_55_P2081432	XR_002130	Gm8953	"2-cell-stage, variable group, member 3-like pseudogene"	2.285
A_55_P2207255	NM_016911	Srpx	sushi-repeat-containing protein	2.328
A_55_P2046877	NM_008239	Foxq1	forkhead box Q1	2.305
A_51_P112355	NM_018738	Igtp	interferon gamma induced GTPase	2.301
A_51_P116651	NM_019759	Dpt	dermatopontin	2.301
A_55_P2113498	NM_010654	Klrd1	"killer cell lectin-like receptor, subfamily D, member 1"	2.291
A_55_P2052016	NM_030209	Crispld2	cysteine-rich secretory protein LCCL domain containing 2	2.358
A_55_P2083879	NM_020518	Vsig2	V-set and immunoglobulin domain containing 2	2.322
A_55_P1979491	NM_007642	Cd28	CD28 antigen	2.259
A_55_P2004016	NM_030209	Crispld2	cysteine-rich secretory protein LCCL domain containing 2	2.289
A_55_P2051039	NM_001099346	Gm11937	predicted gene 11937	2.371
A_51_P510891	NM_007423	Afp	alpha fetoprotein	2.279
A_55_P2122618	AK002412	Asb11	ankyrin repeat and SOCS box-containing 11	2.32
A_51_P126067	NM_013486	Cd2	CD2 antigen	2.307
A_55_P2060243	NM_145467	Itgbl1	"integrin, beta-like 1"	2.266
A_55_P1977782	NM_001081209	Prdm14	PR domain containing 14	1.958
A_55_P2057936	NM_001135115	Gm12250	predicted gene 12250	2.223
A_51_P437240	NM_007929	Emp2	epithelial membrane protein 2	2.277
A_55_P2072035	NM_009382	Thy1	"thymus cell antigen 1, theta"	2.233
A_55_P1978681	NM_146010	Tspan8	tetraspanin 8	2.253
A_55_P1992849	NM_013462	Adrb3	"adrenergic receptor, beta 3"	2.267
A_55_P1989658	NM_013797	Slco1a1	"solute carrier organic anion transporter family, member 1a1"	2.235
A_55_P2017710	NM_033608	Igsf9	"immunoglobulin superfamily, member 9"	2.384
A_51_P246317	NM_008630	Mt2	metallothionein 2	2.219
A_55_P2472435	NM_018734	Gbp3	guanylate binding protein 3	2.229
A_55_P2035946	NM_001002927	Penk	preproenkephalin	2.115
A_55_P1961499	NM_010741	Ly6c1	"lymphocyte antigen 6 complex, locus C1"	2.209
A_55_P2124498	NM_019583	Il17rb	interleukin 17 receptor B	2.241
A_55_P1982404	NM_001177956	Gpm6b	glycoprotein m6b	2.196
A_51_P255456	NM_009994	Cyp1b1	"cytochrome P450, family 1, subfamily b, polypeptide 1"	2.209
A_52_P327664	NM_153564	Gbp5	guanylate binding protein 5	2.195
A_55_P1953788	NM_010583	Itk	IL2-inducible T-cell kinase	2.198

Table S2. (continued)

A_51_P193146	NM_028595	Ms4a6c	"membrane-spanning 4-domains, subfamily A, member 6C"	2.189
A_52_P84027	NM_007824	Cyp7a1	"cytochrome P450, family 7, subfamily a, polypeptide 1"	2.181
A_52_P71686	NM_175406	Atp6v0d2	"ATPase, H+ transporting, lysosomal V0 subunit D2"	2.138
A_55_P1955308	NM_001002898	Sirpb1a	signal-regulatory protein beta 1A	2.175
A_51_P137336	NM_009864	Cdh1	cadherin 1	2.224
A_51_P380309	NM_001113204	Ncam1	neural cell adhesion molecule 1	2.123
A_55_P2184013	NM_175219	C130026I21Rik	RIKEN cDNA C130026I21 gene	2.189
A_66_P103232	ENSMUST00000103366	LOC100046973	"similar to [Human Ig rearranged gamma chain mRNA, V-J-C region and com	2.176
A_51_P480602	NM_009363	Tff2	trefoil factor 2 (spasmolytic protein 1)	2.169
A_55_P2092310	AK195427	NA	NA	2.205
A_51_P338262	NM_011619	Tnnt2	"troponin T2, cardiac"	2.228
A_51_P233160	NM_027309	Lysmd2	"LysM, putative peptidoglycan-binding, domain containing 2"	2.157
A_66_P139666	NM_177013	Tmem229a	transmembrane protein 229A	2.151
A_55_P1991214	NM_001113474	Lair1	leukocyte-associated Ig-like receptor 1	2.134
A_52_P343306	NM_010378	H2-Aa	"histocompatibility 2, class II antigen A, alpha"	2.144
A_51_P123625	NM_008392	Irg1	immunoresponsive gene 1	2.192
A_52_P546162	ENSMUST00000103355	NA	NA	2.098
A_52_P366047	NM_145938	Rpp40	ribonuclease P 40 subunit (human)	2.142
A_55_P1974367	NM_010174	Fabp3	"fatty acid binding protein 3, muscle and heart"	2.114
A_55_P1978994	ENSMUST00000103472	NA	NA	2.147
A_52_P476431	NM_021292	Evc	Ellis van Creveld gene homolog (human)	2.092
A_55_P1958480	BC089308	LOC545005	hypothetical protein LOC545005	2.129
A_55_P1994042	NM_001139519	Zbp1	Z-DNA binding protein 1	2.108
A_55_P2023912	NA	NA	NA	2.241
A_55_P1980601	NM_172701	Oscp1	organic solute carrier partner 1	2.104
A_55_P1954116	NM_133882	C8b	"complement component 8, beta polypeptide"	2.107
A_51_P326191	NM_009251	Serpina3g	"serine (or cysteine) peptidase inhibitor, clade A, member 3G"	2.103
A_52_P299771	NM_007535	Bcl2a1c	B-cell leukemia/lymphoma 2 related protein A1c	2.111
A_55_P1986341	NR_002858	Gm4956	predicted gene 4956	2.093
A_55_P2143041	NM_016966	Phgdh	3-phosphoglycerate dehydrogenase	2.048
A_55_P2154933	NM_207244	Cd200r4	CD200 receptor 4	2.05
A_55_P1997003	BC031891	Serpina4-ps1	"serine (or cysteine) peptidase inhibitor, clade A, member 4, pseudogene 1"	2.097
A_55_P1973995	ENSMUST00000077710	NA	NA	2.137
A_51_P323180	NM_172777	Gbp9	guanylate-binding protein 9	2.129
A_55_P2000595	NA	NA	NA	2.101
A_51_P492830	NM_021886	Cenph	centromere protein H	2.073

Table S2. (continued)

A_52_P244193	NM_009846	Cd24a	CD24a antigen	2.045
A_55_P2124976	NM_145890	Grhl1	grainyhead-like 1 (Drosophila)	2.051
A_55_P1955305	NM_001002898	Sirpb1a	signal-regulatory protein beta 1A	2.068
A_51_P204740	NM_133654	Cd34	CD34 antigen	2.081
A_55_P2040485	XM_889011	Ms4a4a	"membrane-spanning 4-domains, subfamily A, member 4A"	2.007
A_51_P414879	ENSMUST00000103304	NA	NA	2.192
A_55_P2041444	NM_001110784	Xlr3a	X-linked lymphocyte-regulated 3A	2.11
A_55_P2178578	NM_145403	Tmprss4	"transmembrane protease, serine 4"	2.043
A_55_P1978424	NM_007536	Bcl2a1d	B-cell leukemia/lymphoma 2 related protein A1d	2.05
A_51_P302358	NM_008518	Ltb	lymphotoxin B	2.079
A_55_P2033041	NM_001173460	Sirpb1b	signal-regulatory protein beta 1B	2.037
A_55_P2266178	BC028446	AI790276	expressed sequence AI790276	2.024
A_51_P360492	NM_008567	Mcm6	"minichromosome maintenance deficient 6 (MIS5 homolog, S. pombe) (S. ce	2.032
A_55_P1965101	NM_001081643	Xlr3b	X-linked lymphocyte-regulated 3B	2.136
A_52_P590154	NM_013786	Hsd17b6	hydroxysteroid (17-beta) dehydrogenase 6	2.052
A_52_P250644	XM_001479095	LOC100047902	similar to RIKEN cDNA 1700022C21 gene	2.212
A_55_P1964648	NM_001037719	Btla	B and T lymphocyte associated	1.968
A_55_P1962918	NM_001033450	Mnda	myeloid cell nuclear differentiation antigen	2.019
A_51_P299149	NM_145451	Gpx6	glutathione peroxidase 6	2.016
A_51_P394814	NM_022814	Svep1	"sushi, von Willebrand factor type A, EGF and pentraxin domain containing 1	2.003
A_55_P1985544	NM_029911	Kcnk10	"potassium channel, subfamily K, member 10"	2.088
A_52_P410685	NM_033073	Krt7	keratin 7	2.111
A_55_P2022211	NM_026162	Plxdc2	plexin domain containing 2	2.064
A_51_P161021	NM_008332	Ifit2	interferon-induced protein with tetratricopeptide repeats 2	2.076
A_52_P257625	NM_023612	Esm1	endothelial cell-specific molecule 1	2.016
A_51_P108459	NM_008152	Gpr65	G-protein coupled receptor 65	2.032
A_55_P2039541	NM_153820	Arhgap15	Rho GTPase activating protein 15	2.007
A_55_P2033780	NM_010174	Fabp3	"fatty acid binding protein 3, muscle and heart"	1.983
A_51_P519251	NM_019738	Nupr1	nuclear protein 1	1.983
A_52_P390127	AK138801	Klrc1	"killer cell lectin-like receptor subfamily C, member 1"	1.948
A_51_P209818	NM_011178	Prtn3	proteinase 3	1.954
A_55_P2114953	NM_011909	Usp18	ubiquitin specific peptidase 18	1.982
A_55_P1970105	NM_013589	Ltbp2	latent transforming growth factor beta binding protein 2	1.998

Table S3. 173 genes that were under-expressed in the liver of the Nrf2 knock-out mice compared to the wild-type mice after 180 days on high-fat diet.

Probe ID	Primary Accession	Gene Symbol	Gene Name	fold change
A_51_P411271	NM_010902	Nfe2l2	"nuclear factor, erythroid derived 2, like 2"	-1098
A_52_P562267	NM_001033819	9130409I23Rik	RIKEN cDNA 9130409I23 gene	-30.27
A_55_P2032946	NM_008181	Gsta1	"glutathione S-transferase, alpha 1 (Ya)"	-21.27
A_55_P2114342	ENSMUST00000100!	NA	NA	-13.35
A_55_P2017116	XM_001480391	LOC625953	similar to Glutathione S-transferase Ya chain (GST class-alpha) (Ya1)	-12.64
A_51_P253481	NM_021456	Ces1	carboxylesterase 1	-11.38
A_52_P669922	NM_032541	Hamp	hepcidin antimicrobial peptide	-10.74
A_55_P2130885	NM_008552	Mas1	MAS1 oncogene	-9.942
A_55_P2102065	NM_001122660	Gm10639	predicted gene 10639	-9.891
A_52_P289091	NM_007813	Cyp2b13	"cytochrome P450, family 2, subfamily b, polypeptide 13"	-9.87
A_66_P104980	NM_177919	Tceal5	transcription elongation factor A (SII)-like 5	-9.749
A_55_P2126850	NM_021431	Nudt11	nudix (nucleoside diphosphate linked moiety X)-type motif 11	-9.572
A_55_P2164075	XM_001479672	LOC100048172	hypothetical protein LOC100048172	-9.388
A_51_P346893	NM_019578	Extl1	exostoses (multiple)-like 1	-8.162
A_51_P206235	NM_028051	Slc39a5	"solute carrier family 39 (metal ion transporter), member 5"	-7.851
A_55_P2034517	ENSMUST00000156(	NA	NA	-7.58
A_51_P326685	NM_176920	Lrtm1	leucine-rich repeats and transmembrane domains 1	-7.062
A_55_P2387915	AK143789	Gm10804	predicted gene 10804	-6.867
A_55_P2154377	XM_911133	Gm7169	predicted gene 7169	-5.979
A_51_P446512	ENSMUST00000094	2310016G11Rik	RIKEN cDNA 2310016G11 gene	-5.608
A_55_P2047809	NM_175692	Snhg11	small nucleolar RNA host gene 11 (non-protein coding)	-5.605
A_51_P267933	NM_175692	Snhg11	small nucleolar RNA host gene 11 (non-protein coding)	-5.479
A_55_P1975989	NM_172864	Wdr63	WD repeat domain 63	-5.224
A_55_P1966432	NM_010358	Gstm1	"glutathione S-transferase, mu 1"	-5.138
A_55_P2198983	AI606402	4930415C11Rik	RIKEN cDNA 4930415C11 gene	-5.036
A_51_P424338	NM_008706	Nqo1	"NAD(P)H dehydrogenase, quinone 1"	-4.925
A_51_P386648	NM_027227	Glod5	glyoxalase domain containing 5	-4.812
A_55_P2093704	NM_008579	Meig1	meiosis expressed gene 1	-4.718
A_55_P2062190	NM_010358	Gstm1	"glutathione S-transferase, mu 1"	-4.709
A_55_P2160128	NM_001033210	Pls1	plastin 1 (I-isoform)	-4.574
A_51_P514319	NM_172892	Slc13a4	"solute carrier family 13 (sodium/sulfate symporters), member 4"	-4.408
A_51_P199168	NM_007702	Cidea	"cell death-inducing DNA fragmentation factor, alpha subunit-like effector A"	-4.372
A_55_P1985623	NM_029600	Abcc3	"ATP-binding cassette, sub-family C (CFTR/MRP), member 3"	-4.36
A_55_P2171413	NM_008615	Me1	"malic enzyme 1, NADP(+)-dependent, cytosolic"	-4.322
A_55_P2029111	XM_001479090	LOC100048011	hypothetical protein LOC100048011	-4.313

Table S3. (continued)

A_55_P2172396	NM_178796	A530064D06Rik	RIKEN cDNA A530064D06 gene	-4.294
A_66_P124420	NAP001637-001	NA	NA	-4.262
A_51_P471458	NM_020564	Sult5a1	"sulfotransferase family 5A, member 1"	-4.209
A_55_P2016842	NM_008615	Me1	"malic enzyme 1, NADP(+)-dependent, cytosolic"	-4.141
A_51_P343350	NM_033603	Amn	amnionless	-4.039
A_55_P2268221	NM_030113	Arhgap10	Rho GTPase activating protein 10	-4.018
A_55_P2065231	NM_010359	Gstm3	"glutathione S-transferase, mu 3"	-4.016
A_55_P1985346	XM_001475143	Gm13017	predicted gene 13017	-3.998
A_55_P2124712	NM_145603	Ces2	carboxylesterase 2	-3.868
A_65_P02177	NM_026764	Gstm4	"glutathione S-transferase, mu 4"	-3.86
A_55_P1992986	XM_979688	LOC675135	"similar to amyotrophic lateral sclerosis 2 (juvenile) chromosome region, candida	-3.822
A_51_P333111	NM_009676	Aox1	aldehyde oxidase 1	-3.785
A_52_P318361	NM_145603	Ces2	carboxylesterase 2	-3.742
A_55_P2170454	NM_008182	Gsta2	"glutathione S-transferase, alpha 2 (Yc2)"	-3.666
A_55_P2150876	NM_175370	Als2cr12	"amyotrophic lateral sclerosis 2 (juvenile) chromosome region, candidate 12 (hur	-3.614
A_52_P627269	NM_198171	BC015286	cDNA sequence BC015286	-3.568
A_55_P2408588	NM_007489	Arntl	aryl hydrocarbon receptor nuclear translocator-like	-3.552
A_55_P2148171	NM_001168500	A330049M08Rik	RIKEN cDNA A330049M08 gene	-3.512
A_51_P397934	NM_130455	Grin3b	"glutamate receptor, ionotropic, NMDA3B"	-3.503
A_55_P2135153	NM_153558	Lcn13	lipocalin 13	-3.492
A_55_P2093705	NM_008579	Meig1	meiosis expressed gene 1	-3.482
A_55_P2095039	NM_001168500	A330049M08Rik	RIKEN cDNA A330049M08 gene	-3.476
A_55_P2129449	NM_020565	Sult3a1	"sulfotransferase family 3A, member 1"	-3.465
A_52_P578634	NM_033612	Cela1	"chymotrypsin-like elastase family, member 1"	-3.44
A_51_P311958	NM_013623	Orm3	orosomucoid 3	-3.388
A_52_P920158	NM_001081425	Rbm24	RNA binding motif protein 24	-3.359
A_55_P2004452	NM_025703	Tceal8	transcription elongation factor A (SII)-like 8	-3.337
A_52_P602847	NM_008134	Glycam1	glycosylation dependent cell adhesion molecule 1	-3.296
A_55_P2204491	AI131754	AI131754	expressed sequence AI131754	-3.263
A_55_P2124233	NM_177723	Vsig8	V-set and immunoglobulin domain containing 8	-3.263
A_55_P2102060	NM_010359	Gstm3	"glutathione S-transferase, mu 3"	-3.167
A_55_P2069969	NM_001122660	Gm10639	predicted gene 10639	-3.166
A_55_P1972448	XM_001472595	Gm7300	predicted gene 7300	-3.117
A_55_P2100155	NM_001038609	Mapt	microtubule-associated protein tau	-3.114
A_55_P1967350	NM_172881	Ugt2b35	"UDP glucuronosyltransferase 2 family, polypeptide B35"	-3.074
A_55_P1994112	NM_013661	Sema5b	"sema domain, seven thrombospondin repeats (type 1 and type 1-like), transmer	-3.016

Table S3. (continued)

A_52_P9703	NM_009997	Cyp2a4	"cytochrome P450, family 2, subfamily a, polypeptide 4"	-3
A_55_P2007381	ENSMUST0000032[illegible]	NA	NA	-2.932
A_55_P1973844	NM_013661	Sema5b	"sema domain, seven thrombospondin repeats (type 1 and type 1-like), transmen	-2.916
A_55_P1997585	XM_286199	Cyp2a21-ps	"cytochrome P450, family 2, subfamily a, polypeptide 21, pseudogene"	-2.904
A_55_P2052824	NM_001033042	Lrrc14b	leucine rich repeat containing 14B	-2.89
A_51_P327585	NM_026764	Gstm4	"glutathione S-transferase, mu 4"	-2.885
A_55_P2100149	NM_001038609	Mapt	microtubule-associated protein tau	-2.873
A_66_P136778	NM_177922	Mapk15	mitogen-activated protein kinase 15	-2.873
A_55_P1953341	NM_026323	Wfdc2	WAP four-disulfide core domain 2	-2.852
A_55_P1976744	XM_001478262	Gm4324	predicted gene 4324	-2.851
A_55_P2051596	XM_001479558	Gm4499	predicted gene 4499	-2.846
A_51_P163578	NM_172881	Ugt2b35	"UDP glucuronosyltransferase 2 family, polypeptide B35"	-2.843
A_55_P1960828	NM_172371	Slc16a13	"solute carrier family 16 (monocarboxylic acid transporters), member 13"	-2.84
A_55_P2003053	NM_010024	Dct	dopachrome tautomerase	-2.805
A_55_P2007326	NM_009997	Cyp2a4	"cytochrome P450, family 2, subfamily a, polypeptide 4"	-2.783
A_55_P1961466	NM_010024	Dct	dopachrome tautomerase	-2.773
A_55_P1954486	NM_001038609	Mapt	microtubule-associated protein tau	-2.764
A_55_P2126940	XM_001474703	Gm7262	predicted gene 7262	-2.759
A_55_P2051796	NM_001024841	Gm128	predicted gene 128	-2.755
A_55_P2049771	XM_001480935	Gm4621	predicted gene 4621	-2.742
A_55_P2185178	ENSMUST0000096[illegible]	Tmem28	transmembrane protein 28	-2.736
A_55_P1957918	NM_001135192	Asap2	"ArfGAP with SH3 domain, ankyrin repeat and PH domain 2"	-2.725
A_55_P2071906	ENSMUST00000108[illegible]	NA	NA	-2.699
A_51_P276943	NM_016696	Gpc1	glypican 1	-2.69
A_51_P392967	NM_053253	Zmynd10	"zinc finger, MYND domain containing 10"	-2.69
A_55_P1995135	NM_027195	Casz1	"castor homolog 1, zinc finger (Drosophila)"	-2.686
A_51_P481159	NM_173047	Cbr3	carbonyl reductase 3	-2.684
A_51_P156955	NM_013459	Cfd	complement factor D (adipsin)	-2.663
A_52_P278581	BC029005	Zbtb1	zinc finger and BTB domain containing 1	-2.655
A_51_P387400	NM_026535	Serpina12	"serine (or cysteine) peptidase inhibitor, clade A (alpha-1 antiproteinase, antitryp	-2.642
A_51_P362066	NM_007695	Chi3l1	chitinase 3-like 1	-2.636
A_55_P1954842	XM_001477923	LOC100047186	hypothetical protein LOC100047186	-2.629
A_55_P2016166	NM_026323	Wfdc2	WAP four-disulfide core domain 2	-2.614
A_55_P2044653	NM_009999	Cyp2b10	"cytochrome P450, family 2, subfamily b, polypeptide 10"	-2.565
A_55_P1998811	XM_001476688	Gm3430	predicted gene 3430	-2.557
A_55_P2022128	NM_016782	Cntnap1	contactin associated protein-like 1	-2.551

Table S3. (continued)

A_51_P104718	NM_001010941	Gpr12	G-protein coupled receptor 12	-2.539
A_55_P1972436	NM_178666	Themis	thymocyte selection associated	-2.523
A_55_P1997651	NM_207687	Espn	espin	-2.514
A_55_P1998656	NM_001026214	Entpd5	ectonucleoside triphosphate diphosphohydrolase 5	-2.482
A_55_P2058189	NM_029875	Slc35e3	"solute carrier family 35, member E3"	-2.482
A_55_P2090060	NM_172295	BC037703	cDNA sequence BC037703	-2.474
A_51_P358256	NM_009803	Nr1i3	"nuclear receptor subfamily 1, group I, member 3"	-2.465
A_51_P178772	NM_144930	AU018778	expressed sequence AU018778	-2.453
A_51_P324633	NM_007703	Elovl3	"elongation of very long chain fatty acids (FEN1/Elo2, SUR4/Elo3, yeast)-like 3"	-2.427
A_55_P2006008	NM_025429	Serpinb1a	"serine (or cysteine) peptidase inhibitor, clade B, member 1a"	-2.422
A_55_P2101776	NM_001008533	Adora1	adenosine A1 receptor	-2.42
A_55_P2391534	AK015487	4930459C07Rik	RIKEN cDNA 4930459C07 gene	-2.407
A_51_P164630	NM_026808	Fitm1	fat storage-inducing transmembrane protein 1	-2.406
A_55_P2002578	NM_010145	Ephx1	"epoxide hydrolase 1, microsomal"	-2.394
A_55_P1998651	NM_001026214	Entpd5	ectonucleoside triphosphate diphosphohydrolase 5	-2.39
A_55_P1962766	NM_001085513	Fam131c	"family with sequence similarity 131, member C"	-2.374
A_55_P1985351	NM_028060	Slc35f2	"solute carrier family 35, member F2"	-2.374
A_51_P501844	NM_175475	Cyp26b1	"cytochrome P450, family 26, subfamily b, polypeptide 1"	-2.37
A_51_P324351	NM_013900	Mfi2	antigen p97 (melanoma associated) identified by monoclonal antibodies 133.2 ar	-2.365
A_52_P327156	NR_027970	0610008F07Rik	RIKEN cDNA 0610008F07 gene	-2.336
A_55_P2035824	NM_009803	Nr1i3	"nuclear receptor subfamily 1, group I, member 3"	-2.306
A_55_P2006236	NM_009466	Ugdh	UDP-glucose dehydrogenase	-2.296
A_55_P1975080	NM_009467	Ugt2b5	"UDP glucuronosyltransferase 2 family, polypeptide B5"	-2.283
A_51_P377856	NM_133994	Gstt3	"glutathione S-transferase, theta 3"	-2.24
A_51_P401184	NM_001164763	Rarres1	retinoic acid receptor responder (tazarotene induced) 1	-2.232
A_55_P2061338	NM_153081	Slc16a11	"solute carrier family 16 (monocarboxylic acid transporters), member 11"	-2.223
A_55_P2002577	NM_010145	Ephx1	"epoxide hydrolase 1, microsomal"	-2.22
A_55_P2318584	NM_007474	Aqp8	aquaporin 8	-2.217
A_52_P256914	NM_010000	Cyp2b9	"cytochrome P450, family 2, subfamily b, polypeptide 9"	-2.215
A_51_P495269	NM_008508	Lor	loricrin	-2.21
A_51_P302856	NM_053215	Ugt2b37	"UDP glucuronosyltransferase 2 family, polypeptide B37"	-2.197
A_55_P2157620	ENSMUST00000099	NA	NA	-2.194
A_66_P106611	NM_008137	Gna14	"guanine nucleotide binding protein, alpha 14"	-2.184
A_52_P883557	NM_001033286	Slc30a10	"solute carrier family 30, member 10"	-2.176
A_52_P469502	NM_028176	Cda	cytidine deaminase	-2.172
A_55_P1991416	XM_001473368	NA	NA	-2.171

Table S3. (continued)

A_51_P246677	NM_020002	Rec8	REC8 homolog (yeast)	-2.168
A_51_P365019	NM_010295	Gclc	"glutamate-cysteine ligase, catalytic subunit"	-2.165
A_55_P2078955	NM_007474	Aqp8	aquaporin 8	-2.156
A_52_P661587	NM_001025576	Ccdc141	coiled-coil domain containing 141	-2.151
A_52_P315976	NM_009416	Tpm2	"tropomyosin 2, beta"	-2.15
A_55_P2143219	NM_011242	Rasgrp2	"RAS, guanyl releasing protein 2"	-2.144
A_51_P372550	NM_026770	Cgref1	cell growth regulator with EF hand domain 1	-2.138
A_52_P536494	NM_008709	Mycn	"v-myc myelocytomatosis viral related oncogene, neuroblastoma derived (avian)'	-2.134
A_55_P2042156	NM_007377	Aatk	apoptosis-associated tyrosine kinase	-2.102
A_55_P2021572	NM_001164284	C87414	expressed sequence C87414	-2.09
A_55_P2010906	NM_153405	Rbm45	RNA binding motif protein 45	-2.087
A_55_P2029203	NM_026700	Dopey2	dopey family member 2	-2.084
A_51_P315682	NM_183029	Igf2bp2	insulin-like growth factor 2 mRNA binding protein 2	-2.08
A_51_P200083	NM_023168	Grina	"glutamate receptor, ionotropic, N-methyl D-aspartate-associated protein 1 (gluta	-2.078
A_52_P21486	NM_183257	Hamp2	hepcidin antimicrobial peptide 2	-2.077
A_55_P2096043	NM_025590	Acot11	acyl-CoA thioesterase 11	-2.076
A_51_P498882	NM_010001	Cyp2c37	"cytochrome P450, family 2. subfamily c, polypeptide 37"	-2.071
A_51_P404193	NM_022435	Sp5	trans-acting transcription factor 5	-2.065
A_51_P369784	NM_133660	Es22	esterase 22	-2.064
A_55_P2105858	NM_030693	Atf5	activating transcription factor 5	-2.053
A_55_P2143233	NM_011242	Rasgrp2	"RAS, guanyl releasing protein 2"	-2.051
A_51_P372141	NM_001039509	Pnkd	paroxysmal nonkinesiogenic dyskinesia	-2.05
A_55_P1994132	AK148454	Tmem184a	transmembrane protein 184a	-2.032
A_52_P413947	NM_010840	Mthfr	"5,10-methylenetetrahydrofolate reductase"	-2.02
A_55_P2180744	NM_153508	Clstn3	calsyntenin 3	-2.008
A_55_P2173817	NM_008552	Mas1	MAS1 oncogene	-2.008
A_55_P1960291	NM_013769	Tjp3	tight junction protein 3	-2.006
A_55_P2033272	NM_021481	Treh	trehalase (brush-border membrane glycoprotein)	-2.005
A_52_P142520	NM_198292	Tex2	testis expressed gene 2	-1.989
A_52_P456561	NM_007435	Abcd1	"ATP-binding cassette, sub-family D (ALD), member 1"	-1.958
